# Adsorption and Aggregation Activity of Sodium Dodecyl Sulfate and Rhamnolipid Mixture

**DOI:** 10.1007/s11743-016-1916-6

**Published:** 2016-12-10

**Authors:** Diana Mańko, Anna Zdziennicka, Bronisław Jańczuk

**Affiliations:** Department of Interfacial Phenomena, Faculty of Chemistry, Maria Curie-Skłodowska University, Maria Curie-Skłodowska Sq. 3, 20-031 Lublin, Poland

**Keywords:** Surfactant, Biosurfactant, Adsorption, Micellization, Gibbs free energy of adsorption, Gibbs free energy of micellization

## Abstract

Measurements of the surface tension, density and viscosity of sodium dodecyl sulfate (SDS) and rhamnolipid (RL) mixtures were carried out in aqueous solution. From the obtained results, composition of mixed surface layer at the water–air interface, mixed micelles, parameter of intermolecular interactions, activity of SDS and RL in the surface layer and micelles, Gibbs standard free energy of adsorption and micellization as well as Gibbs free energy of SDS and RL mixing in the surface layer and micelles were established. These parameters were discussed in the light of independent adsorption of SDS and RL and the size of their molecules as well as the area in contact with water molecules. A correlation between the number of water molecules in contact with those of SDS and RL and standard free energy of adsorption as well as micellization of these surfactants was observed. A correlation between the apparent and partial molar volumes of RL and SDS in their mixture and size of surfactant molecules as well as the average distance between molecules was also found. The parameter of intermolecular interactions indicates that there is a synergetic effect in the reduction of water surface tension and micelle formation.

**Electronic supplementary material:**

The online version of this article (doi:10.1007/s11743-016-1916-6) contains supplementary material, which is available to authorized users.

## Introduction

Surfactants are amphiphilic compounds which have very wide application due to their ability to reduce surface tension, increase solubility of many substances connected with micelle formation or detergency power [1, 2]. Two fundamental properties of surfactants; the tendency to adsorb at the interfaces, and to form micellar aggregates are connected with changes in water structure if an aqueous solution is applied [1, 2]. The number of water molecules which are in contact with molecules of surfactant and their orientation and interaction with particular groups of surfactant molecules dictates the adsorption and aggregation properties of the surfactant.

In many practical applications, a mixture of different kinds of surfactants is used [1, 2]. Carefully selection of the mixture components allows for optimal conditions in a given process because adsorption and aggregation properties of a mixture can be significantly different from its components. In many cases, the surfactant mixture exhibits synergy in surface tension reduction and formation of mixed micelles [1, 3–5]. The synergetic effect, which is strongest in anionic and cationic surfactant mixtures, can even occur in mixtures of two surfactants of the same kind [1, 6]. The synergetic effect is often explained on the basis of intermolecular interactions in the mixed monolayer and micelles [1, 7]. These parameters based on the mole fraction of the surfactants in the monolayer and micelle are determined by application of regular solutions theory [1, 7–9]. They do not provide information about the kind of intermolecular interactions which are connected with the particular groups present in the surfactant molecules. The kind of groups in the surfactant molecules is decisive with regard to the size and contactable area with water molecules as well as the orientation connected with the kind of intermolecular interactions.

Thus, to explain accurately the synergetic effect of the surfactant mixture in the reduction of water surface tension and micelle formation, the size of the surfactant, its contactable area with water molecules, the kinds of functional groups in the structure of surfactant molecule, the relative mole fraction of the surfactants in the mixed monolayer and the fraction of the surface occupied by the surfactant mixture and individual components should be known. To achieve such information, the measurements of surface tension, density and viscosity of aqueous surfactant mixtures at constant concentration of one surfactant while changing the other can be helpful.

Recently special attention has been paid to biosurfactants which are produced by microorganisms and have very interesting properties [10]. They have very high surface activity, low critical micelle concentration, are resistant to temperature and salinity changes, are nontoxic and biodegradable [11]. Unfortunately, despite these advantages, biosurfactants are not widely used because of their high production costs. However, it seems possible that the addition of the biosurfactant to synthetic surface active agents considerably improves the mixture properties. One of the most studied classes of biosurfactants are rhamnolipids. The main emphasis of research has been on use, antibacterial properties and production rather than on physical and chemical effects, especially in combination with other surfactants [12–15]. In the literature there are many examples of binary surfactant mixtures, but they are composed of classical synthetic surfactants [1, 3–7]. It seems to be interesting to obtain information about the behavior of mixtures of classical surfactants with biosurfactants. Thus, the purpose of this study was to investigate volumetric and surface properties of rhamnolipid mixtures with a classical anionic surfactant. Sodium dodecyl sulfate was chosen as a synthetic surfactant due to the fact that this is a component of many commonly applied products [1, 2]. The aim of this study was to determine surface tension, density and viscosity of aqueous solutions of rhamnolipid and sodium dodecyl sulfate mixture at constant concentration of one surfactant as well as the thermodynamic analysis based on the size of these surfactants and their contactable area with water molecules.

## Experimental

### Materials

**Fig. 1:**
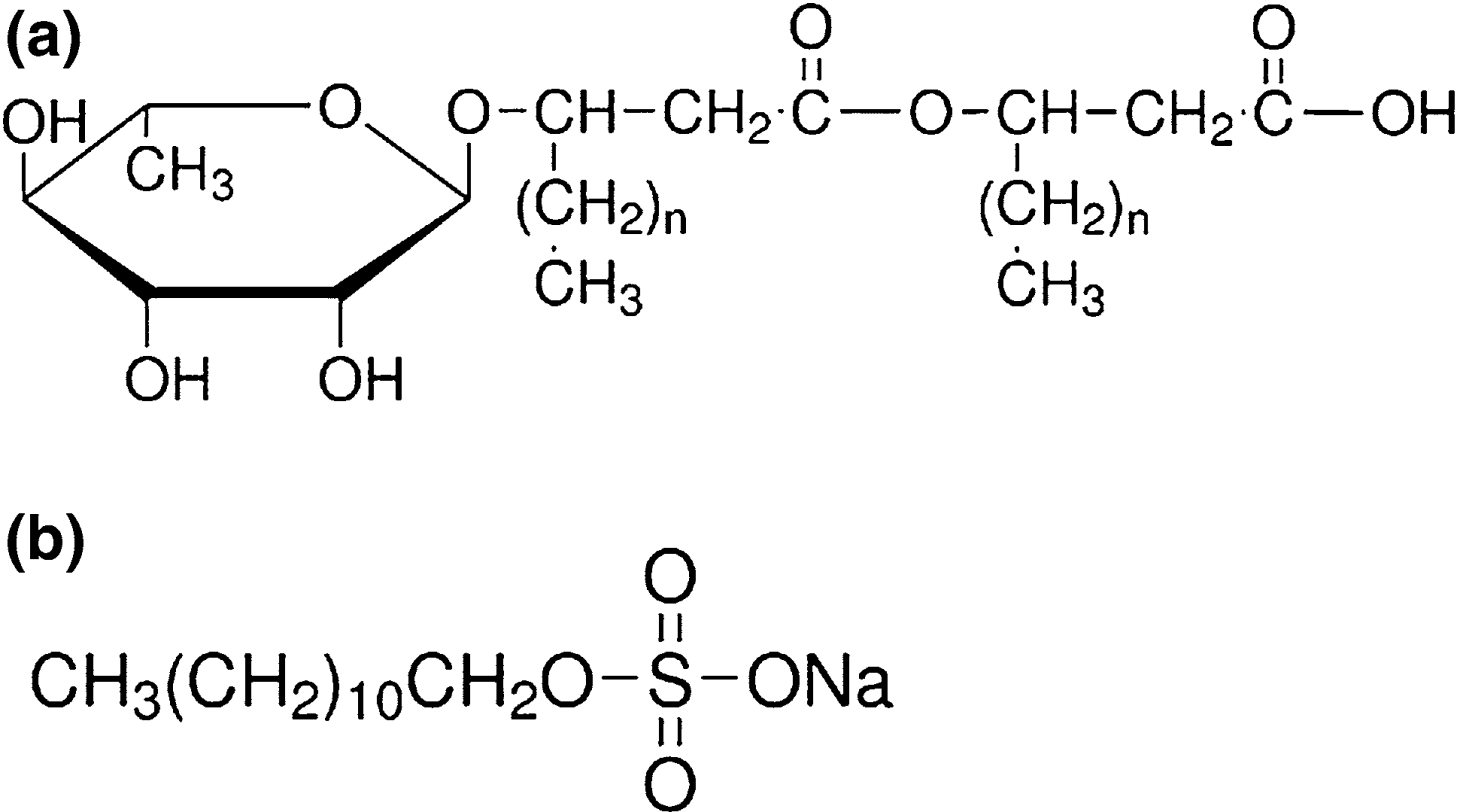
Structures of RL (**a**) and SDS (**b**) molecules

R-95 rhamnolipid (RL) and sodium dodecyl sulfate (SDS) were purchased from Sigma-Aldrich and were used without further purification. The surfactant structures are shown in Fig. 1. The series of aqueous solutions of RL and SDS mixture were prepared at constant concentration of one surfactant. The RL concentration (*C*_RL_) was in the range from 0.0002 to 40 mg/dm^3^, SDS from 1 × 10^−8^ to 1 × 10^−2^ M. All solutions were prepared using doubly distilled and deionized water (Destamat Bi18E) with an internal specific resistance equal to 18.2 MΩ.

### Measurements

The equilibrium surface tension (*γ*_LV_) of the aqueous solutions of RL and SDS mixtures was measured by the Krüss K100C surface tensiometer using the du Noüy ring detachment method as well as a Wilhelmy plate. Before the measurements, the platinum ring and plate were cleaned with distilled water and heated to a red color. The average value of the surface tension for each mixture was obtained on the basis of more than 10 measurements. The standard deviation depending on the methods and surfactant concentration was in the range from ±0.1 to ±0.25 mN/m.

The density of the studied solutions was measured using a densitometer DMA 5000 Anton Paar. The precision of the density measurements given by the manufacturer is ±0.000001 g/m^3^.

The dynamic viscosity measurements of the aqueous solutions of RL and SDS mixture were performed with the Anton Paar viscosimeter (AMVn) with a precision of 0.0001 mPa·s.

All the experiments were carried out at 293 K within ±0.1 K.

### Thermodynamic Consideration

During the adsorption and aggregation processes of surfactants at constant temperature and pressure, the changes of Gibbs free energy of the system are observed, which satisfies Eq. 1 [16]:

1 $$\Delta G = \Delta H - T\Delta S$$

where Δ*H* and Δ*S* are the changes of the enthalpy and entropy at a given temperature (*T*), respectively.

The changes of Gibbs free energy of aqueous solutions of surfactants expressed by Eq. (1) are connected with the standard Gibbs free energy of adsorption (Δ*G*_ads_^o^) and micellization (Δ*G*_mic_^o^).

The standard Gibbs free energy of adsorption of the surface active ion or molecule at the water–air interface results from the transfer of hydrophobic part of surfactant from water to air phase and changes of hydration degree of hydrophilic part of surfactant. Thus, it was suggested that this energy can be expressed by Eq. 2 [17]:

2 $$\Delta G_{\text{ads}}^{\text{o}} = \left( {\gamma_{\text{T}} - \gamma_{\text{TW}} } \right)A_{\text{T}} N + \left( {\gamma_{{{\text{WH}}_{1} }} - \gamma_{\text{WH}} } \right)A_{\text{H}} N$$

where *γ*_T_ is the surface free energy of the tail, *γ*_TW_ is the interfacial free energy of the water-tail, *A*_*T*_ is the contactable area of the surfactant tail or its part, the *γ*_WH_ and *γ*_WH1_ are the surface free energies of water-head at different degrees of head hydration, *A*_H_ is the contactable area of the surfactant head or that with a part of the tail and *N* is the Avogadro number.

In turn, the standard Gibbs free energy of micellization is connected with Gibbs free energy of the interaction (Δ*G*_int_) of the surface active ion or molecule through the water phase. This energy is expressed by Eq. 3 [18]:

3 $$\Delta G_{\text{int}}^{{}} = - 2\gamma_{\text{TW}} A_{\text{T}}^{*} - 1\gamma_{\text{WH}} A_{\text{H}}^{*} + \Delta G_{\text{H}}^{\text{EL}} A_{\text{H}}^{*}$$

where *A*_T_^*^ and *A*_H_^*^ are the contactable areas between the tails and heads of two surfactant molecules or ions, respectively and Δ*G*_H_^EL^ is the Gibbs free energy of  the electrostatic interactions between two ions.

As follows from Eqs. (2) and (3) the contactable area of surfactant molecules or surface active ions plays an important role in their adsorption and aggregation activity. On the other hand, the changes of the Gibbs free energy during adsorption and micellization are related to those of enthalpy and entropy. The changes of enthalpy during these processes are commonly small and come from number of formed hydrogen bonds. However, the changes of entropy are the main forces of the Gibbs surface free energy changes. They are due to reorientation of the water molecules in contact with the surface active ion or molecule. The number of water molecules in contact with the surfactant molecules should determine the entropy changes in the adsorption and micellization processes. Thus, it is possible that knowledge of the contactable area of the hydrophilic and hydrophobic parts of surfactant molecules allows to prediction of the adsorption and aggregation activity of a given surfactant.

To calculate this area it is assumed that the volumes occupied by particular parts of the surfactant molecule are close to that of cubes whose sizes depend on the length of bonds and angle between them. The volume of SDS surface active ion was divided into two cubes; one dealing with C_12_H_25_– and the other with –OSO_3_. In the case of the rhamnolipid, the volume was expressed by four cubes dealing with the four functional groups. In the rhamnolipid there are two CH_3_(CH_2_)_6_– groups, C_6_O_4_H_6_– and –CHCH_2_COOCHCH_2_COOH. Using this approach, the calculated contactable area of SDS and RL is equal to 429.24 and 721.12 Å^3^, respectively. If we assume that one water molecule occupies 10 Å^2^, then at the first approximation, 72 molecules of water can come in contact with one surface of the RL and 43 with SDS. The ratio of the number of water molecules contacted with RL and SDS molecules is equal to 1.67. The Gibbs standard free energy of mono-rhamnolipid adsorption calculated from the Langmuir equation is equal to −85.04 kJ/mol [19] and SDS −52.1 kJ/mol [20]. The ratio of free energies is equal to 1.63. On the other hand, the Gibbs free energy of RL micellization is reported in the literature to be equal to −60.92 kJ/mol (average) and −36.2 kJ/mol (average) for SDS. The ratio of energies is equal to 1.68. It means that the ratio of the Gibbs standard free energy of micellization and adsorption of RL to SDS is very close to the ratio of the water molecules in contact with RL and SDS, respectively. Taking into account the structure of rhamnolipid and SDS molecules, the hydrogen bond formation between these ions is not excluded. In such case the number of water molecules contacted with RL and SDS ions can be changed and the repulsive interactions between the hydrophilic parts of surfactants decrease. It should influence on the composition of mixed monolayers and micelles.

### Composition of the RL and SDS Monolayer at the Water–Air Interface

**Fig. 2:**
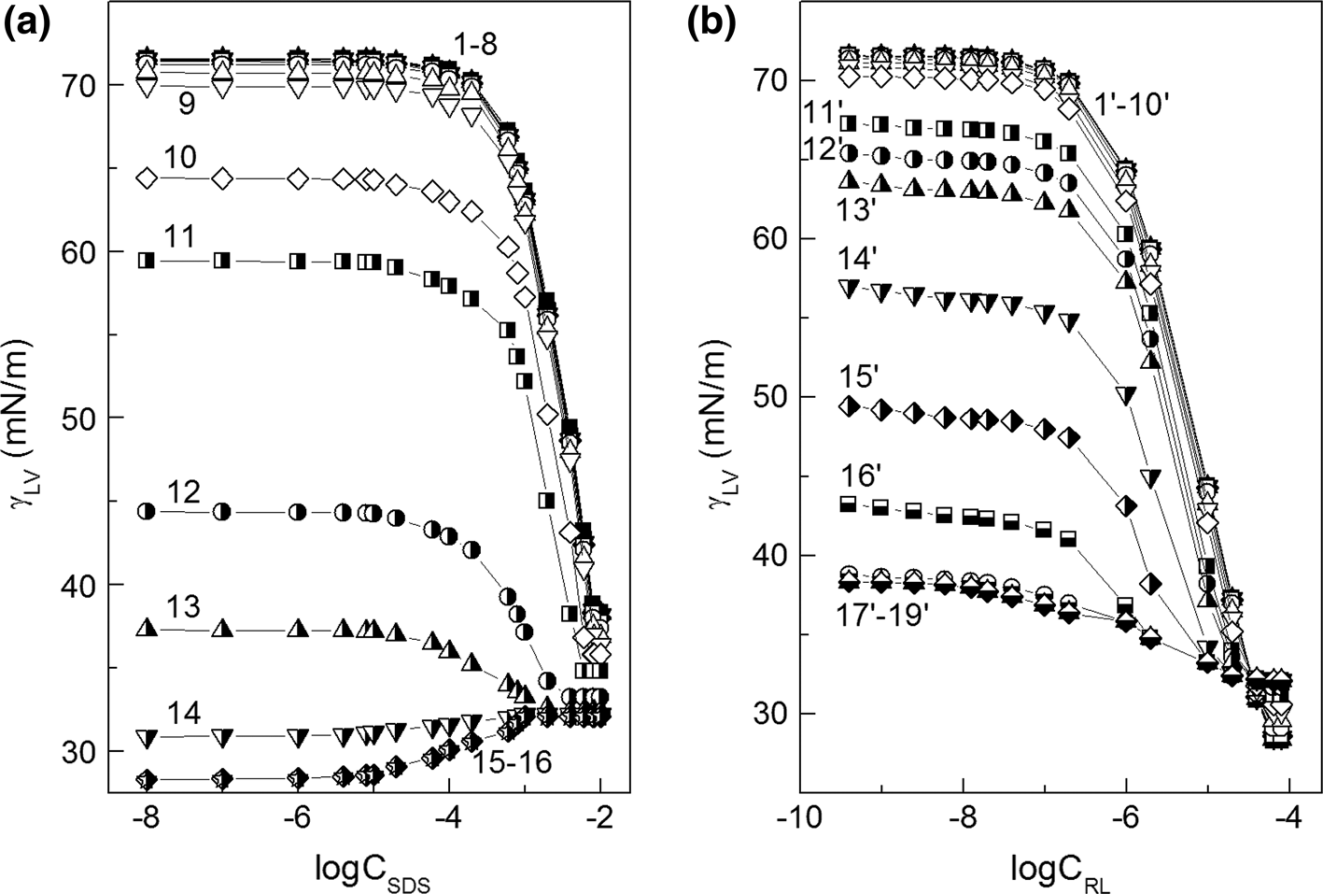
A plot of the surface tension (*γ*_LV_) of aqueous solutions of SDS and RL mixture *vs* the logarithm of surfactant concentration—**a** SDS (*C*_SDS_) and **b** RL (*C*_RL_). Curves 1–16 correspond to the constant RL concentration (*C*_RL_) equal to 3.97 × 10^−10^; 9.92 × 10^−10^; 2.48 × 10^−9^; 5.95 × 10^−9^; 1.24 × 10^−8^; 1.98 × 10^−8^; 3.97 × 10^−8^; 9.92 × 10^−8^; 1.98 × 10^−7^; 9.92 × 10^−7^; 1.98 × 10^−6^; 9.9 × 10^−6^; 1.98 × 10^−5^; 3.97 × 10^−5^; 6.35 × 10^−5^ and 7.94 × 10^−5^ M; curves 1′–19′ to the constant SDS concentration (*C*_SDS_) equal to 1 × 10^−8^; 1 × 10^−7^; 1 × 10^−6^; 4 × 10^−6^; 8 × 10^−6^; 1 × 10^−5^; 2 × 10^−5^; 6 × 10^−5^; 1 × 10^−4^; 2 × 10^−4^; 6 × 10^−4^; 8 × 10^−4^; 1 × 10^−3^; 2 × 10^−3^; 4 × 10^−3^; 6 × 10^−3^, 8 × 10^−3^, 8.25 × 10^−3^ and 1 × 10^−2^ M, respectively

**Fig. 3:**
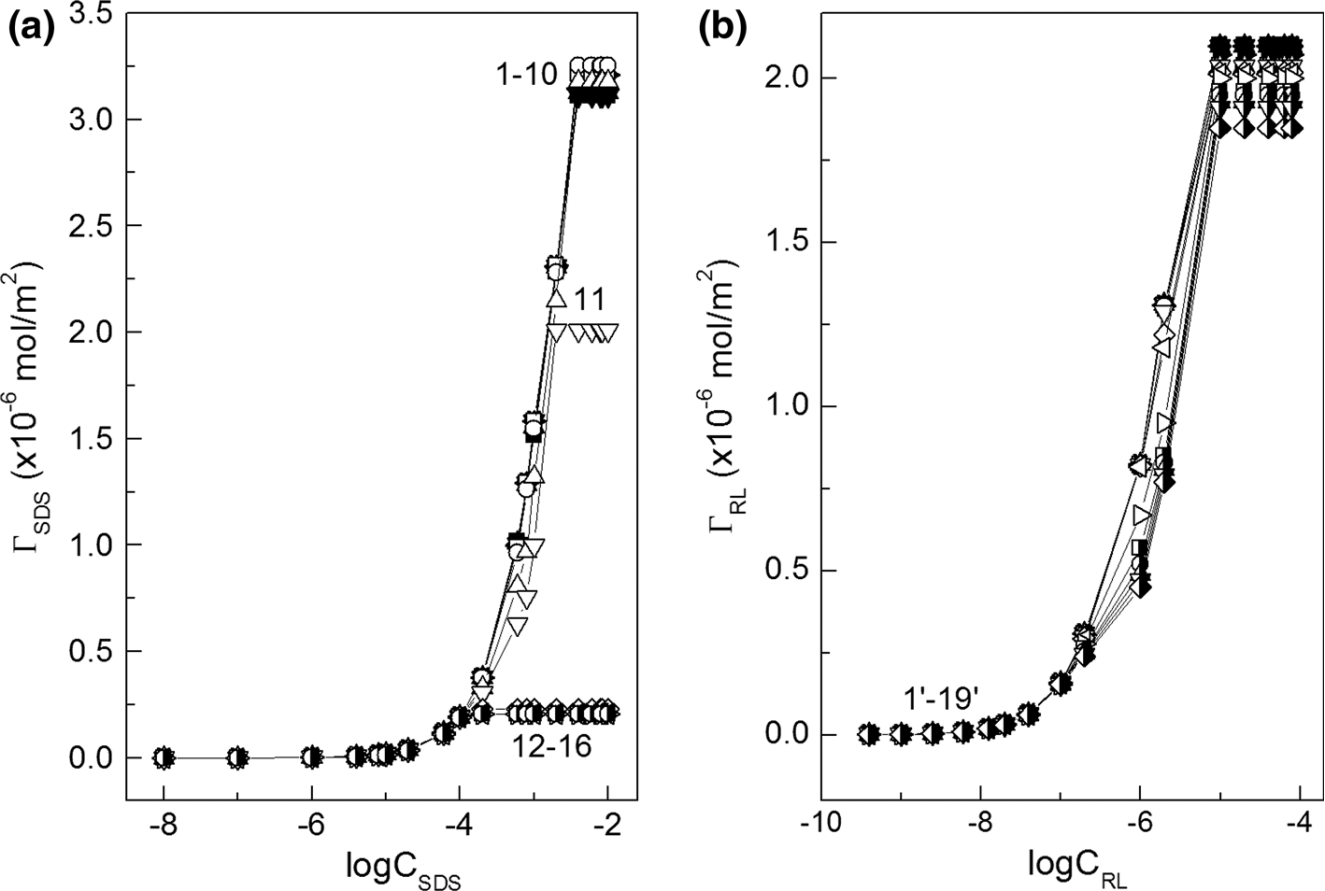
A plot of the Gibbs surface excess concentration of SDS (*Γ*_SDS_) calculated from Eq. S1a and RL (*Γ*_RL_) calculated from Eq. S1b *vs* the logarithm of surfactant concentration—**a** SDS (*C*_SDS_) and **b** RL (*C*_RL_). See Fig. 2 for the description of curves 1–16 and 1′–19′

The composition of the mixed monolayer at the water–air interface strongly depends on the activity of particular components in the layer. This activity decides not only about the composition of the surface layer but also about the fraction of interface occupied by surfactant molecules. The composition of the layer can be established on the basis of Hua and Rosen as well as Rubingh theory [1, 21, 22]. However, to determine the fraction of the interface occupied by surfactant molecules, the Gibbs surface excess concentration should be known. In the case of SDS and rhamnolipid which belong to anionic surfactants, their Gibbs surface excess concentrations (*Γ*_SDS_ and *Γ*_RL_, respectively) can be determined from the surface tension isotherms at the constant concentration of one surfactant (Fig. 2a, b) and the Gibbs isotherm equation (Eqs. S1a and S1b). The shape of the *Γ*_SDS_ and *Γ*_RL_ isotherms (Fig. 3a, b) is similar to those of an individual surfactant in the absence of another. However, the maximal Gibbs surface excess concentration of SDS is practically constant in the range of RL concentration corresponding to the unsaturated monolayer at the water–air interface of individual RL (Fig. 3a) [19]. On the other hand, the same relation is observed for RL (Fig. 3b) [20]. More information about the mutual influence of RL and SDS on its adsorption at the water–air interface can be provided from the total Gibbs surface excess concentration (*Γ*_SDS_ + *Γ*_RL_) (Fig. S1). As can be seen the maximum of the total surface excess concentration is observed.

**Fig. 4:**
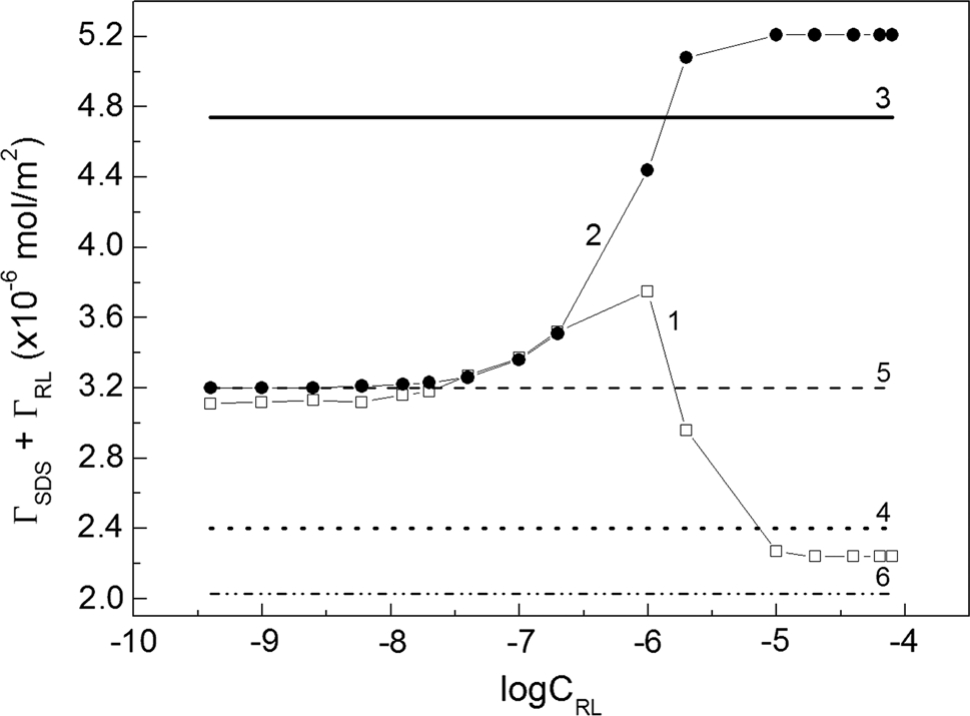
A plot of the maximal total Gibbs surface excess concentration of SDS and RL mixture (*Γ*_SDS_ + *Γ*_RL_) taken from Fig. S1 (curve 1) and the Gibbs surface excess concentration calculated from Eq. S1c using this excess for individual surfactants (curve 2) *vs* the logarithm of RL concentration (*C*_RL_). The lines correspond to the limiting Gibbs surface excess concentration of SDS (line 3, Ref. [20]) and RL (line 4, Ref. [19]) as well as to the maximal Gibbs surface excess concentration of SDS (line 5, Ref. [20]) and RL (line 6, Ref. [19]), respectively

To explain this maximum, change after the addition of RL to the SDS solution was considered (Fig. 4, curve 1). In this Fig. the sum of *Γ*_SDS_ + *Γ*_RL_ for independent adsorption is also presented (curve 2). It is interesting that in the range of RL concentration corresponding to its unsaturated monolayer in the absence of SDS, the “real” sum is nearly the same as the “hypothetical” one (curves 1 and 2). The “real” sum was obtained from the data presented in Fig. 3a, b and the “hypothetical” one on the basis of surface excess concentration of individual surfactants [19, 20]. In the case of “real” isotherm *Γ*_SDS_ + *Γ*_RL_ increases from $\varGamma_{{_{\text{SDS}} }}^{\hbox{max} }$ (line 5) to a maximum at the concentration being close to the first one corresponding to the saturated monolayer of RL in the absence of SDS [19]. This maximum is lower than that of $\varGamma_{{_{\text{SDS}} }}^{\infty }$($\varGamma_{{_{\text{SDS}} }}^{\infty }$ is a limiting Gibbs surface excess concentration of SDS in the absence of RL at the water–air interface) [20]. It means that in the range of RL concentration from 0 to that corresponding to the maximum, there is no influence of SDS on RL adsorption and *vice versa* [19].

Above this concentration, the RL molecules remove those from the mixed surface layer and the sum *Γ*_SDS_ + *Γ*_RL_ decreases to a value somewhat lower than that of the limiting area of the RL molecule ($\varGamma_{{_{\text{RL}} }}^{\infty }$) (curve 4) [19] but higher than the maximum obtained in the absence of SDS (*Γ*_RL_^max^) (curve 6). It should be also pointed out that the changes of “real” *Γ*_SDS_ + *Γ*_RL_ are between the values corresponding to those obtained for the limiting area of studied surfactants. The same conclusion can be drawn if the changes of *Γ*_SDS_ + *Γ*_RL_ are considered as a function of SDS concentration. The independent adsorption of one component from the surfactants mixture takes place if its concentration is lower than that corresponding to the saturated monolayer of the individual surfactant.

This is documented by comparison of the measured and calculated from Eqs. S2 and S3 (Figs. S2–S3) values of surface tension. It appeared that both isotherms of surface tension calculated on the basis of independent adsorption of SDS and RL (Eq. S2) as well as by using the Fainerman and Miller equation (Eq. S3) [23, 24] are nearly the same as those measured if the concentration of one component of surfactant mixture is lower than that corresponding to its saturated monolayer at the water–air interface in the absence of another one. Thus, this comparison confirms the conclusion drawn on the basis of the Gibbs surface excess concentration of RL and SDS at the water–air interface. It means that under the above-mentioned conditions, there is no evident mutual influence of SDS and RL on their adsorption at the water–air interface. As was shown, the number of water molecules in contact with RL ones is higher than with SDS molecules and that the ratio of these numbers is close to that of the Gibbs standard free energy of adsorption of these surfactants.

Thus, the RL tendency to adsorb at the water–air interface is higher than SDS. It means that if the monolayer is saturated by SDS molecules, the RL ones should replace them. However, the addition of RL causes the increase of total adsorption of the surfactants mixture. This increase is observed in the concentration of RL from 0 to the concentration in the bulk phase at which its saturated monolayer in the absence of SDS is formed. This is probably possible because of the decrease in repulsive interactions between the head of RL and SDS in comparison to the RL–RL and SDS–SDS interactions as a result of the hydrogen bond formation between the –OH group in the RL molecule and the oxygen atom of the SDS one. For this reason the values of *Γ*_SDS_ + *Γ*_RL_ under the above-mentioned conditions are higher than the maximal Gibbs surface excess concentration of both individual surfactants [19, 20].

The considerations based on the measured and calculated values of the surface tension of the aqueous solutions of RL and SDS mixture as well *Γ*_SDS_ and *Γ*_RL_ do not clearly explain the mutual influence of RL and SDS on their adsorption at the water–air interface, particularly at the concentration corresponding to the mixed saturated monolayer. For this reason the mole fraction of the area occupied by RL and SDS molecules at the water–air interface (Figs. S4a and S4b) as well as those of these surfactants in the mixed monolayer were determined on the basis of *Γ*_SDS_ and *Γ*_RL_ (Eqs. S4, S5 and S6). If we consider the changes of the area occupied by RL molecules at the water–air interface in the presence of SDS, then we can state that this area is the same as for the solution of individual RL in the range of its concentration lower than that corresponding to its saturated monolayer (Fig. S4b). However, at the RL concentration corresponding to its saturated monolayer in the absence of SDS and the concentration of SDS corresponding to its unsaturated monolayer in the absence of RL, the area occupied by RL molecules (curves 2′ and 3′) is larger than that in the absence of SDS (curve 1′). In the case the concentration of SDS corresponds to its saturated monolayer in the absence of RL, the area occupied by RL molecules (curve 4′) is nearly the same as for the individual RL (curve 1′). Thus, the replacement of SDS molecules by RL ones takes place at the RL concentration corresponding to that when its saturated monolayer in the absence of SDS is formed. The same conclusion can be drawn when the area occupied by SDS is considered (Fig. S4a). On the basis of the changes of the area occupied by RL and SDS molecules, it can be stated that replacement of SDS molecules by RL ones takes place when the concentration of both surfactants is close to the saturated monolayer of individual surfactants.

**Fig. 5:**
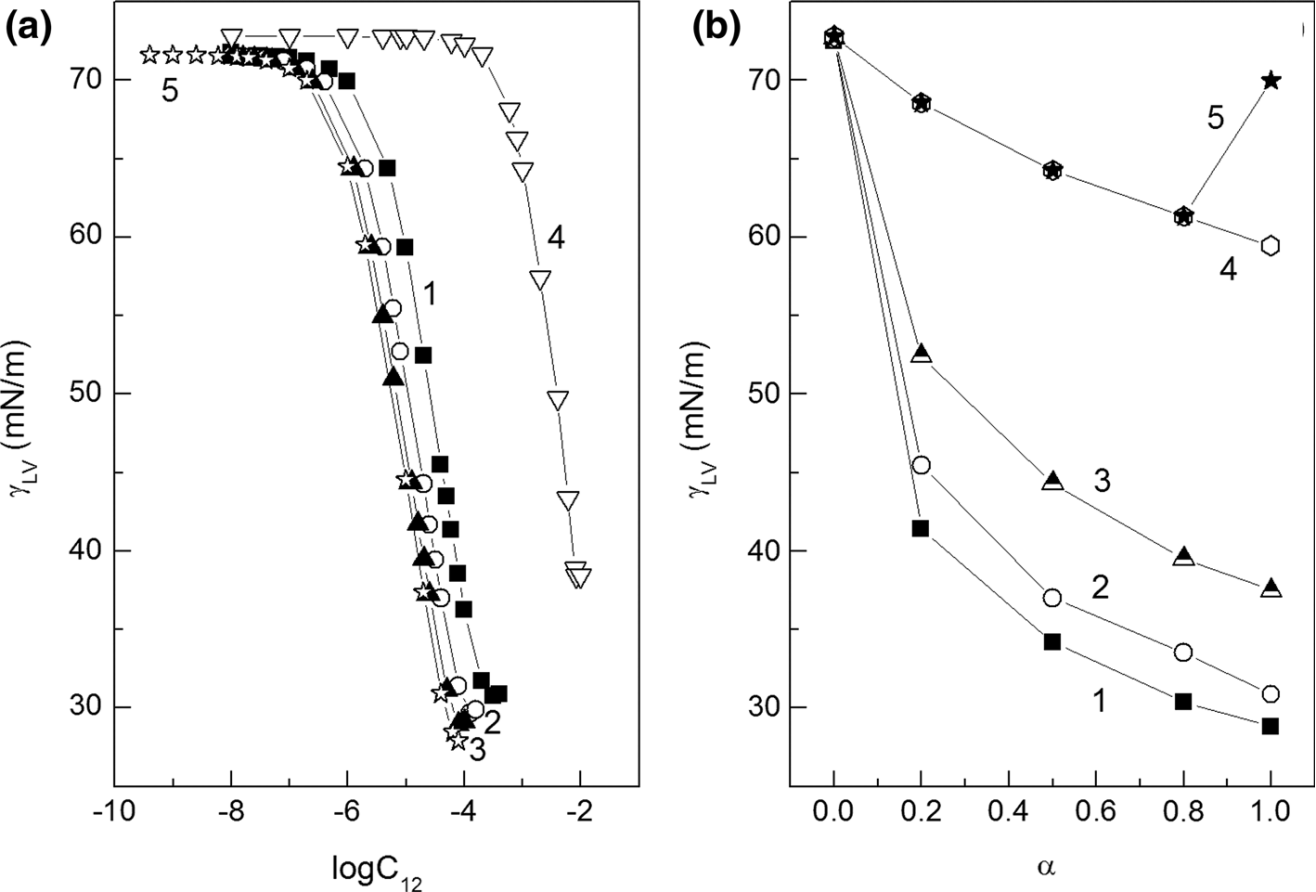
A plot of the surface tension (*γ*_LV_) of aqueous solutions of SDS and RL mixture *vs* the logarithm of its concentration (*C*_12_). Curves 1–3 correspond to the constant value of the RL mole fraction in the mixture equal to 0.2, 0.5 and 0.8, respectively and curves 4 and 5 represent the isotherm of the Gibbs surface excess concentration of SDS and RL taken from the literature [19, 20] (Fig. 5a) and a plot of the changes of the surface tension (*γ*_LV_) of aqueous solution of SDS and RL mixture as a function of mole fraction of RL in the mixture (α) at the concentration of the mixture in the bulk phase equal to 6 × 10^−5^ M (curve 1), 4 × 10^−5^ M (curve 2), 2 × 10^−5^ M (curve 3), 2 × 10^−6^ M (curve 4) and 2 × 10^−7^ M (curve 5) (Fig. 5b)

This is also confirmed by comparison of the summary area occupied by RL and SDS molecules at the water–air interface to those of “hypothetical” ones (Figs. S5, S6). It means that as mentioned above the independent adsorption of SDS and RL takes place if the concentration of one surfactant is lower than that at which its individual statured monolayer starts to form. It can be clearly seen from Fig. S7 in which the mole fraction of RL in the mixed monolayer is presented (calculated from Eqs. S7 and S8). At the concentration of SDS and RL corresponding to the saturated monolayer of individual surfactants, the “real” mole fraction of RL in the mixed monolayer is higher than the “hypothetical” one. It should be emphasized that at high concentration of SDS and RL in the mixture, the mole fraction of RL is close to 1. It is interesting to know the relation between the mole fraction of RL in the mixed monolayer calculated on the basis of the Gibbs surface excess concentration of particular components and that obtained on the basis of Hua, Rosen and Rubingh theory [1, 21, 22]. For this reason we considered the changes of the surface tension of aqueous solutions of SDS and RL mixture as a function of its concentration at mole fractions of RL in the mixture equal to 0.2, 0.5 and 0.8 (Fig. 5a).

**Fig. 6:**
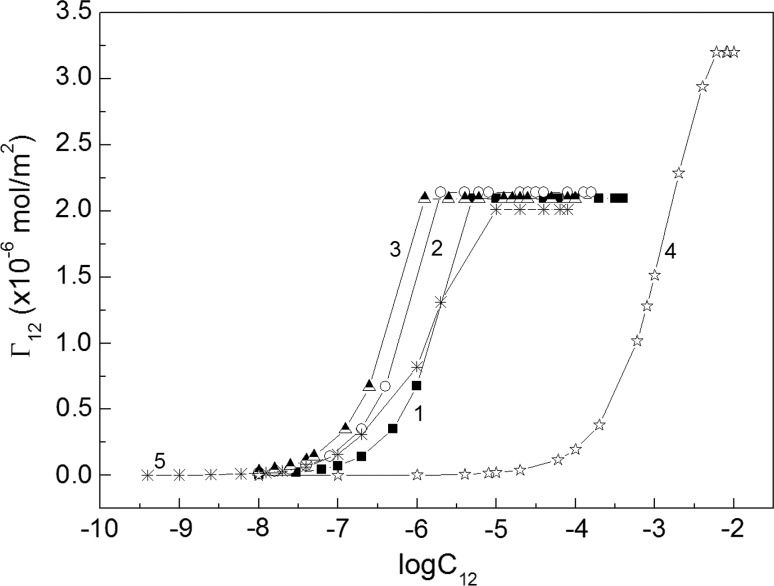
A plot of the Gibbs surface excess concentration of SDS and RL mixture (*Γ*_12_) (Eq. S1c) *vs* the logarithm of its concentration (*C*_12_). Curves 1–3 correspond to the constant value of RL mole fraction in the mixture equal to 0.2, 0.5 and 0.8, respectively and curves 4 and 5 represent the isotherm of the Gibbs surface excess concentration of SDS and RL taken from the literature [19, 20]

On the basis of the data presented in Fig. 5a, the Gibbs surface excess concentration of SDS and RL mixture at the water–air interface (*Γ*_12_) (Eq. S1c) (Fig. 6), the mole fraction of surfactants in the mixed monolayer (*X*_SDS_ and *X*_RL_) (Eq. S9), the parameter of intermolecular interactions (*β* ^*δ*^) (Eq. S10) and the activity coefficients of SDS (*f*_SDS_) (Eq. S11a) and RL (*f*_RL_) (Eq. S11b) in the mixed monolayer at the water–air interface were calculated.

The surfactant mole fraction, parameter of intermolecular interactions and activity coefficients were calculated by applying the Hua, Rosen and Rubingh theory [1, 21, 22] and the surface tension of aqueous solutions of mixture equal to 55, 50, 45 and 40 mN/m. It follows from these calculations that there is a synergetic effect in the water surface tension reduction because the *β* ^*δ*^ parameter is negative and second condition (|*β* ^*δ*^| > | ln (*C*_1_^0^/*C*_2_^0^)|) is also fulfilled. However, the minimum of the dependence between the surface tension and the mole fraction of RL in the mixture is observed only at low concentrations of the mixture (Fig. 5b) but at high concentrations of the mixture only the negative deviation from the linear dependence occurs. It also appeared that the mole fraction of RL in the mixed saturated monolayer calculated on the basis of the Hua, Rosen and Rubingh theory [1, 21, 22] is considerably higher than that in the bulk phase and is close to that calculated on the basis of the Gibbs surface excess concentration of SDS and RL mixture.

### Standard Gibbs Surface Free Energy of Adsorption

The adsorption of surfactants at different interfaces is one of their characteristic properties. The measure of surfactants tendency to adsorb is the standard Gibbs free energy of adsorption (Δ*G*_ads_^0^). This energy can be determined on the basis of many approaches but the Langmuir equation modified by de Boer is very often applied [1, 25] (Eq. S12). Application of this equation in the case of ionic surfactants is correlated with the question whether for the ionic surfactant type 1:1 electrolyte *RT* or 2*RT* should be used. In the literature this problem is still not clearly explained and some authors use *RT* but others 2*RT*. In our opinion it is more correct if *RT* is used, so we calculated Δ*G*_ads_^0^ in this way. From the obtained data it results that (Figs. S8a and S8b), when the concentration of one surfactant corresponds to its unsaturated monolayer at the water-interface in the absence of another, the values of Δ*G*_ads_^0^ are constant. This indicates that there are no interactions between the RL and SDS molecules or these interactions have an insignificant effect on Δ*G*_ads_^0^ values. The values of Δ*G*_ads_^0^ in this concentration range are close to those of Δ*G*_ads_^0^ calculated for individual RL and SDS in the absence of another surfactant at the same assumption [19, 20] which confirms that the RL has no influence on the SDS adsorption at the water–air interface and *vice versa*.

To explain more precisely the mutual influence of RL and SDS on their adsorption at the water–air interface, the systems in which the mole fraction of RL in the mixture with SDS is equal to 0.2; 0.5 and 0.8 (Fig. S9) are taken into account. The values of Δ*G*_ads_^0^ for the surfactant mixture calculated from the Langmuir equation (Eq. S12) (Fig. S9, curve 1) are different from those calculated using the Δ*G*_ads_^0^ values of RL in the absence of SDS and *vice versa* and the mole fraction of surfactants in the mixture in the bulk phase (Eq. S13a) (Fig. S9, curve 4). It means that there is no ideal mixing of RL and SDS in the surface layer and on the basis of Δ*G*_ads_^0^ of the individual SDS and RL, it is impossible to predict Δ*G*_ads_^0^ of the mixture. On the other hand, Δ*G*_ads_^0^ of the mixture should be comparable to that determined on the basis of the RL and SDS mole fraction in the mixed layer (Eq. S13b). Taking into account the mole fraction of these surfactants in the surface layer determined on the basis of the Hua, Rosen and Rubingh theory [1, 21, 22] and Δ*G*_ads_^0^ of individual surfactants, Δ*G*_ads_^0^ of the mixture was determined (Fig. S9, curve 2). It appeared that there are some differences between the Δ*G*_ads_^0^ values determined from the Langmuir equation (Eq. S12) and those based on the mole fraction of surfactants in the monolayer (Eq. S13b). Therefore, Δ*G*_ads_^0^ mixing of RL and SDS in the surface layer was calculated (Eq. S13c) and added to the Gibbs surface free energy of adsorption obtained on the basis of the mole fraction of RL and SDS in the mixed layer (Fig. S9, curve 3). In such case a good agreement between Δ*G*_ads_^0^ values of SDS and RL mixture at the water–air interface obtained in this way and those obtained from the Langmuir equation (Eq. S12) is observed. This confirms our suggestion that there are strong interactions between the SDS and RL molecules in the surface layer.

On the basis of our consideration mentioned above, it can be concluded that the tendency to adsorb of a given surfactant depends on the number of water molecules which are removed from contact with surfactant molecules during the adsorption process. It should depend on whether dehydration of head of surfactants takes place and part of hydrophobic chain which can transfer from a liquid to air phase. It is known that in the micelles only a part of the hydrocarbon tail is not in contact with water molecules. Thus if for example it is assumed that only half of hydrophobic tail of SDS and RL molecules is present in the air phase, then Δ*G*_ads_^0^ calculated from Eq. (2) at the assumption that dehydration of surfactant heads does not occur is equal to −27.22 and −44.61 kJ/mol for SDS and RL, respectively. This example suggests that in the mixed monolayer, the part of head present in the air can change with the composition of the surface layer and therefore the standard Gibbs energy of adsorption does not change linearly as a function of surfactants mixture composition. The ratio of Δ*G*_ads_^0^ of RL to SDS is equal to 1.64 which is comparable to that of the water molecules being in contact with surfactant molecules in water (1.67).

### Critical Micelle Concentration and Standard Gibbs Free Energy of Micellization

**Fig. 7:**
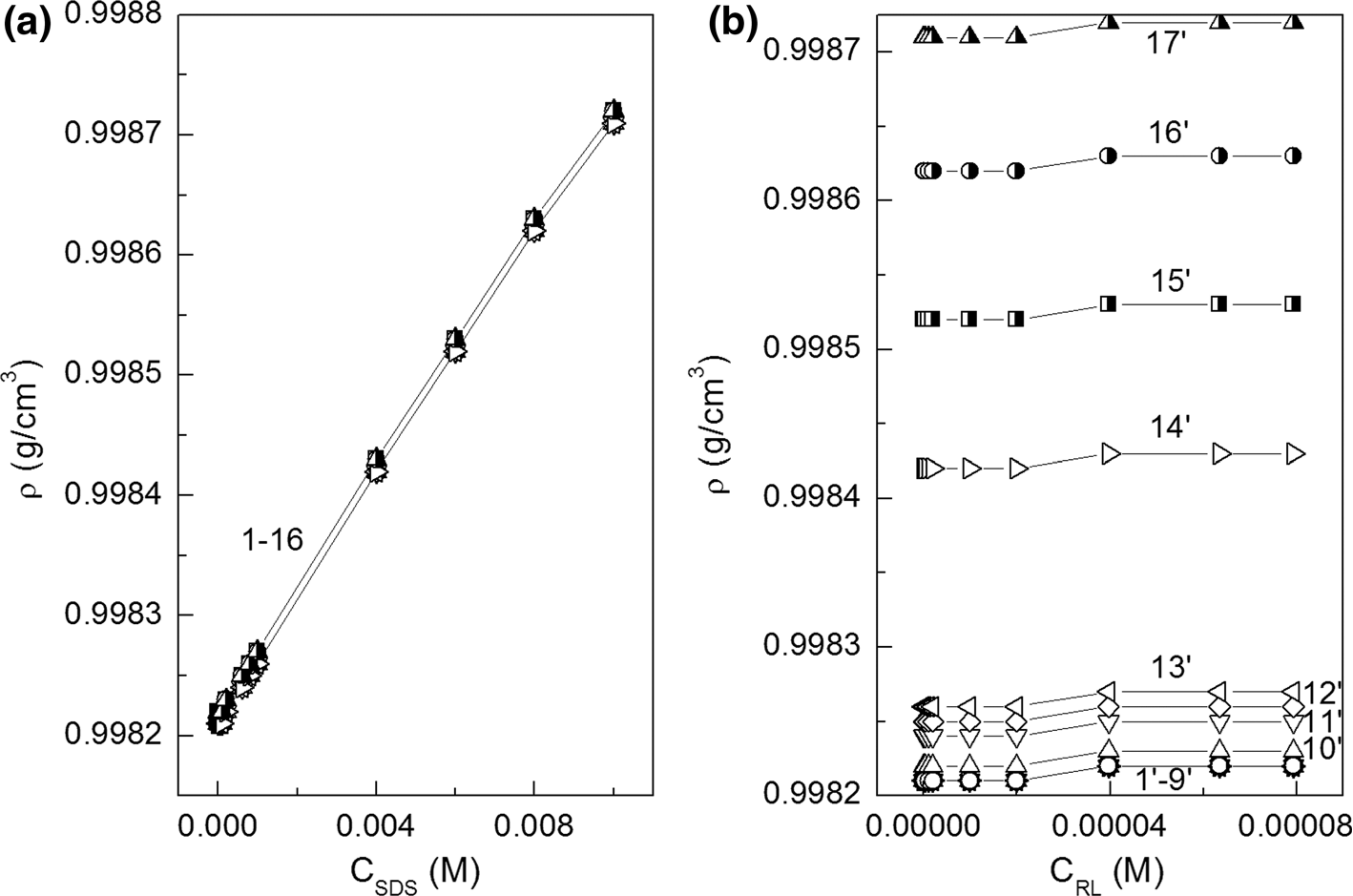
A plot of the density (*ρ*) of aqueous solutions of SDS and RL mixture *vs* surfactant concentration—**a** SDS (*C*_SDS_) and **b** RL (*C*_RL_). See Fig. 2 for the description of curves 1–16. Curves 1′–17′ correspond to the constant SDS concentration equal to 1 × 10^−8^; 1 × 10^−7^; 1 × 10^−6^; 4 × 10^−6^; 8 × 10^−6^; 1 × 10^−5^; 2 × 10^−5^; 6 × 10^−5^; 1 × 10^−4^; 2 × 10^−4^; 6 × 10^−4^; 8 × 10^−4^; 1 × 10^−3^; 4 × 10^−3^; 6 × 10^−3^, 8 × 10^−3^ and 1 × 10^−2^ M, respectively

**Fig. 8:**
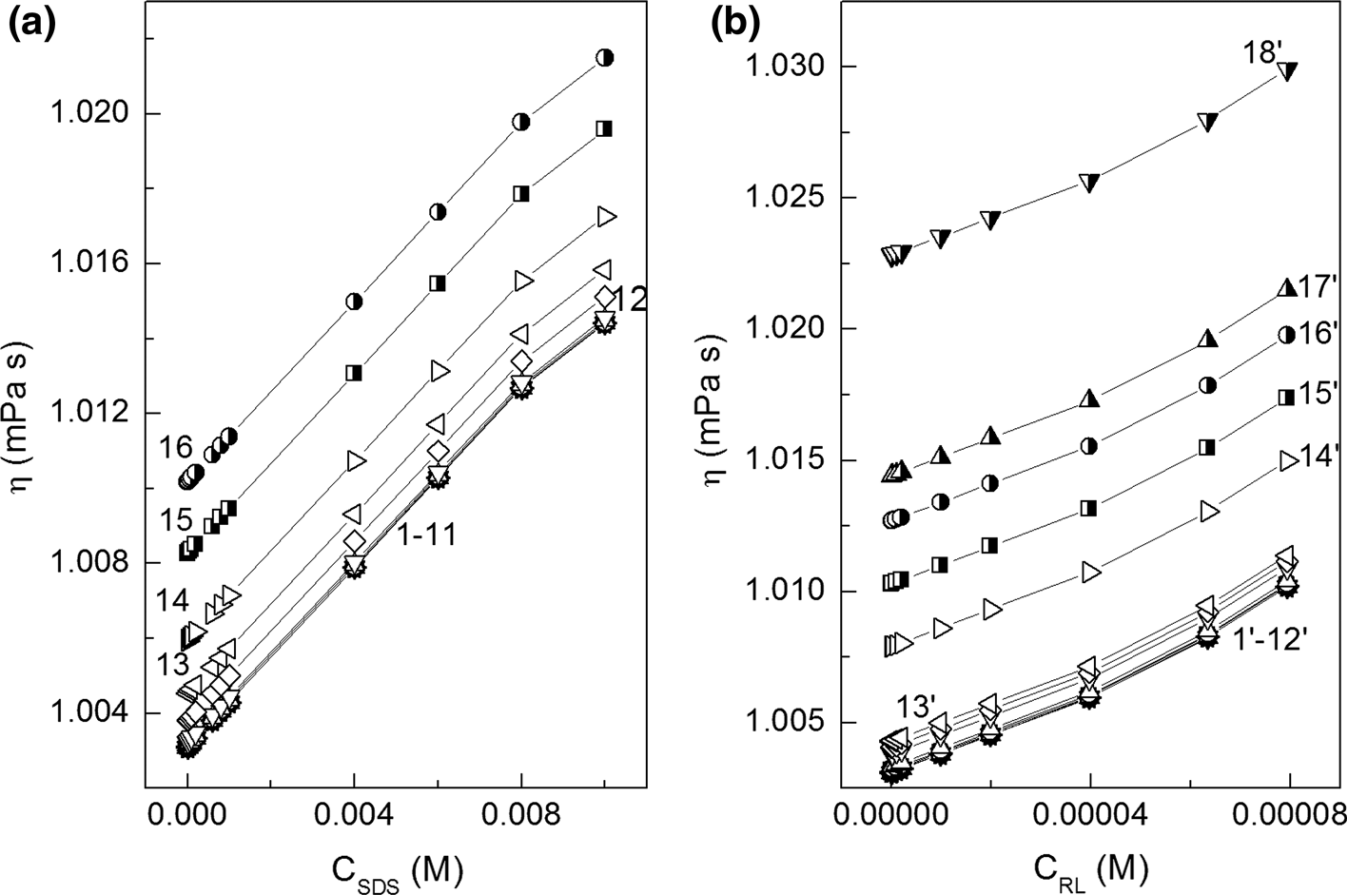
A plot of the dynamic viscosity (*η*) of aqueous solutions of SDS and RL mixture *vs* surfactant concentration—**a** SDS (*C*_SDS_) and **b** RL (*C*_RL_). See Fig. 2 for the description of curves 1–16. Curves 1′–18′ correspond to the constant SDS concentration equal to 1 × 10^−8^; 1 × 10^−7^; 1 × 10^−6^; 4 × 10^−6^; 8 × 10^−6^; 1 × 10^−5^; 2 × 10^−5^; 6 × 10^−5^; 1 × 10^−4^; 2 × 10^−4^; 6 × 10^−4^; 8 × 10^−4^; 1 × 10^−3^; 4 × 10^−3^; 6 × 10^−3^, 8 × 10^−3^, 1 × 10^−2^ and 2 × 10^−2^, respectively

**Fig. 9:**
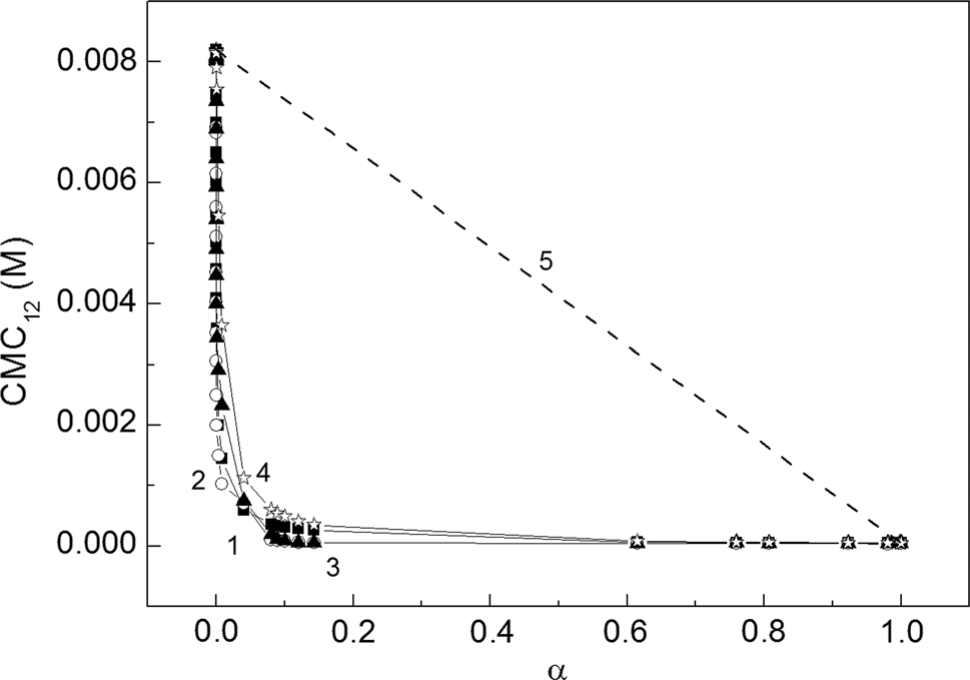
A plot of the CMC values of aqueous solutions of SDS and RL mixture (*CMC*_12_) *vs* the mole fraction of RL in the bulk phase (*α*). Curve 1–3 correspond to the CMC_12_ determined from the isotherm of the surface tension (Fig. 2a, b), density (Fig. 7a, b) and viscosity (Fig. 8a, b), respectively. Curve 4 represents the isotherm of CMC_12_ calculated from Eq. S14 and curve 5 corresponds to the CMC_12_ values calculated from Eq. (4)

The critical micelle concentration (CMC) is the concentration at which surfactants can form micelles. This concentration, for the same surfactant can have somewhat different values depending on the method of its determination. The CMC values of RL and SDS mixtures were determined on the basis of surface tension (Fig. 2a, b), density (Fig. 7a, b) and viscosity isotherms (Fig. 8a, b). The CMC values were also calculated from Eq. S14 which is satisfied for ideal mixing of surfactants in the micelle (Fig. 9, curve 1). It appeared the CMC values determined from the surface tension (Fig. 9, curve 2), density (curve 3) and viscosity (curve 4) isotherms are different from those calculated from Eq. (S14). It means that there is no ideal mixing of SDS and RL in micelles. It is also interesting that above the mole fraction of RL equal to 0.2 in the mixture with SDS in the bulk phase the CMC is nearly the same as for individual RL [19]. To explain the behaviour of SDS and RL in the micelles, the mole fraction of SDS (*X*_SDS_^M^) and RL (*X*_RL_^M^) (Eq. S15), the parameter of intermolecular interactions (*β* ^*M*^) (Eq. S16) as well as the activity coefficients of SDS (*f*_SDS_^M^) Eq. (S17a) and RL (*f*_RL_^M^) (Eq. S17b) in the micelle were determined using the Hua, Rosen and Rubingh theory [1, 21, 22].

**Fig. 10:**
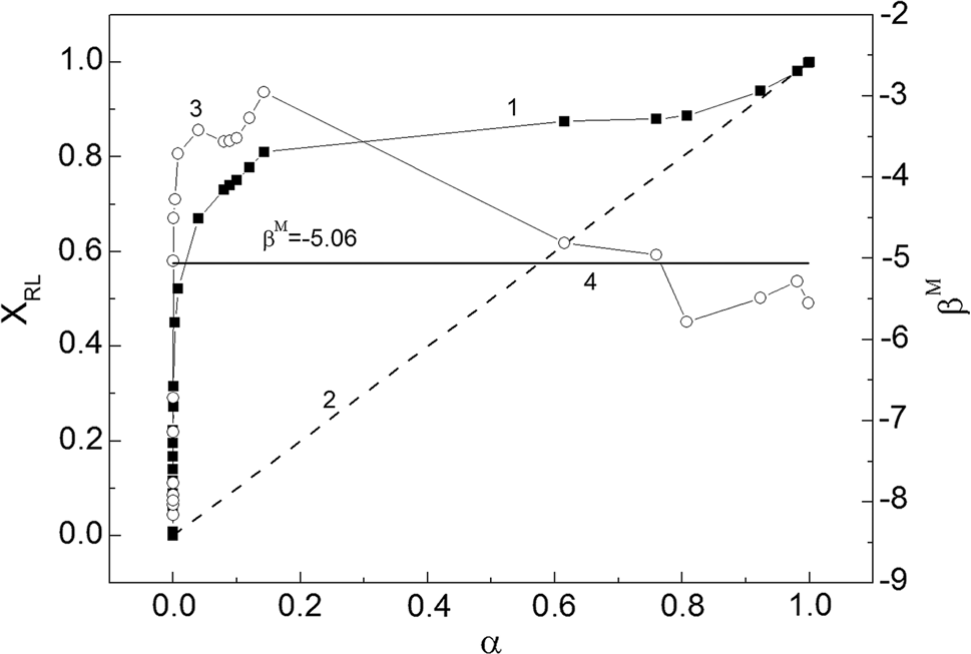
A plot of the RL mole fraction in the mixed micelle (*X*_RL_) (curve 1) and parameter of intermolecular interactions of surfactants in the micelle (*β* ^*M*^) (curve 3) calculated from Eq. S16 *vs* the mole fraction of RL in the mixture with SDS in the bulk phase (*α*). Line 2 refers to the same *X*_RL_ and *α* values and line 4 corresponds to the values of *β* ^*M*^ below which the second condition of the synergetic effect in the mixed micelle formation exists (|*β* ^*M*^| > | ln (*C*_1_^*M*^/*C*_2_^*M*^)|)

As expected the mole fraction of RL in the micelle was higher in comparison to that in the bulk phase (Fig. 10). A considerable increase in the difference between the mole fraction of RL in the micelle in comparison to the bulk phase is observed in the range of *α* from 0 to 0.2 but from 0.2 to 0.8 the mole fraction of RL in the micelle is almost constant and close to 0.8. Knowing the mole fraction of RL and SDS in the mixed micelle, it was possible to calculate the CMC of mixed micelles (CMC_12_) from the following equation [3]:

4 $${\text{CMC}}_{12} = X_{\text{RL}} {\text{CMC}}_{\text{RL}} + (1 - X{}_{\text{RL}})\;{\text{CMC}}_{\text{SDS}}$$

Indeed Eq. (4) is fulfilled in the case when there is ideal mixing of two surfactants in the micelle and then the linear dependence between CMC_12_ and the mole fraction of one component in the mixed micelle exists (Fig. 9). From Fig. 9 we can see that there is a great negative deviation between the CMC_12_ values determined from the surface tension isotherm and those calculated with Eq. (4). However, on the basis of this deviation it is difficult to prove that there is a synergetic effect in the mixed micelle formation of RL and SDS. In the literature two theoretical approaches commonly used for the analysis of the synergetic effect in the mixed micelle formation can be found. The regular solution theory was proposed by Rosen and Rubingh [1, 21, 22] and later, the Bergström theory [3] based on the Poisson–Boltzman equation. Both approaches focus on comparing CMC with the ideal mixture behaviour. Rosen *et al*. proved that there is a synergetic effect in the mixed micelle formation if the parameter of intermolecular interactions in the micelle (*β* ^*M*^) is negative. In our case the *β* ^*M*^ parameter is negative in the whole range of RL mole fraction in the bulk phase (Fig. 10). However, this condition is necessary but not sufficient to state that there is a synergetic effect in the micelle formation. The second condition must be fulfilled if the synergetic effect exists (|*β* ^*M*^| > | ln (*C*_1_^*M*^/*C*_2_^*M*^)|). It appeared that the second condition is fulfilled only in the range of *α* below 10^−4^ and above 0.8 (Fig. 10).

On the other hand, Bergström and Eriksson [3] in order to show the synergetic effect for the mixtures of two ionic surfactants with identical head groups but different hydrocarbon tails derived the following equation:

5 $$\begin{aligned} CMC_{{12}} (X_{{RL}} ) = &\; X_{{{\text{RL}}}} \frac{{\exp \left[ {\frac{{\left( { - \left( {1 - X_{{{\text{RL}}}} } \right)} \right)\left( {1 - \lambda } \right)}}{{X_{{{\text{RL}}}} + \lambda \left( {1 - X_{{{\text{RL}}}} } \right)}}} \right]}}{{X_{{{\text{RL}}}} + \lambda \left( {1 - X_{{{\text{RL}}}} } \right)}}{\text{CMC}}_{{{\text{RL}}}} \\ & \quad + \left( {1 - X_{{{\text{RL}}}} } \right)\frac{{\lambda \exp \left[ {\frac{{X_{{{\text{RL}}}} \left( {1 - \lambda } \right)}}{{X_{{{\text{RL}}}} + \lambda \left( {1 - X_{{{\text{RL}}}} } \right)}}} \right]}}{{X_{{{\text{RL}}}} + \lambda \left( {1 - X_{{{\text{RL}}}} } \right)}}{\text{CMC}}_{{{\text{SDS}}}} \\ \end{aligned}$$

**Fig. 11:**
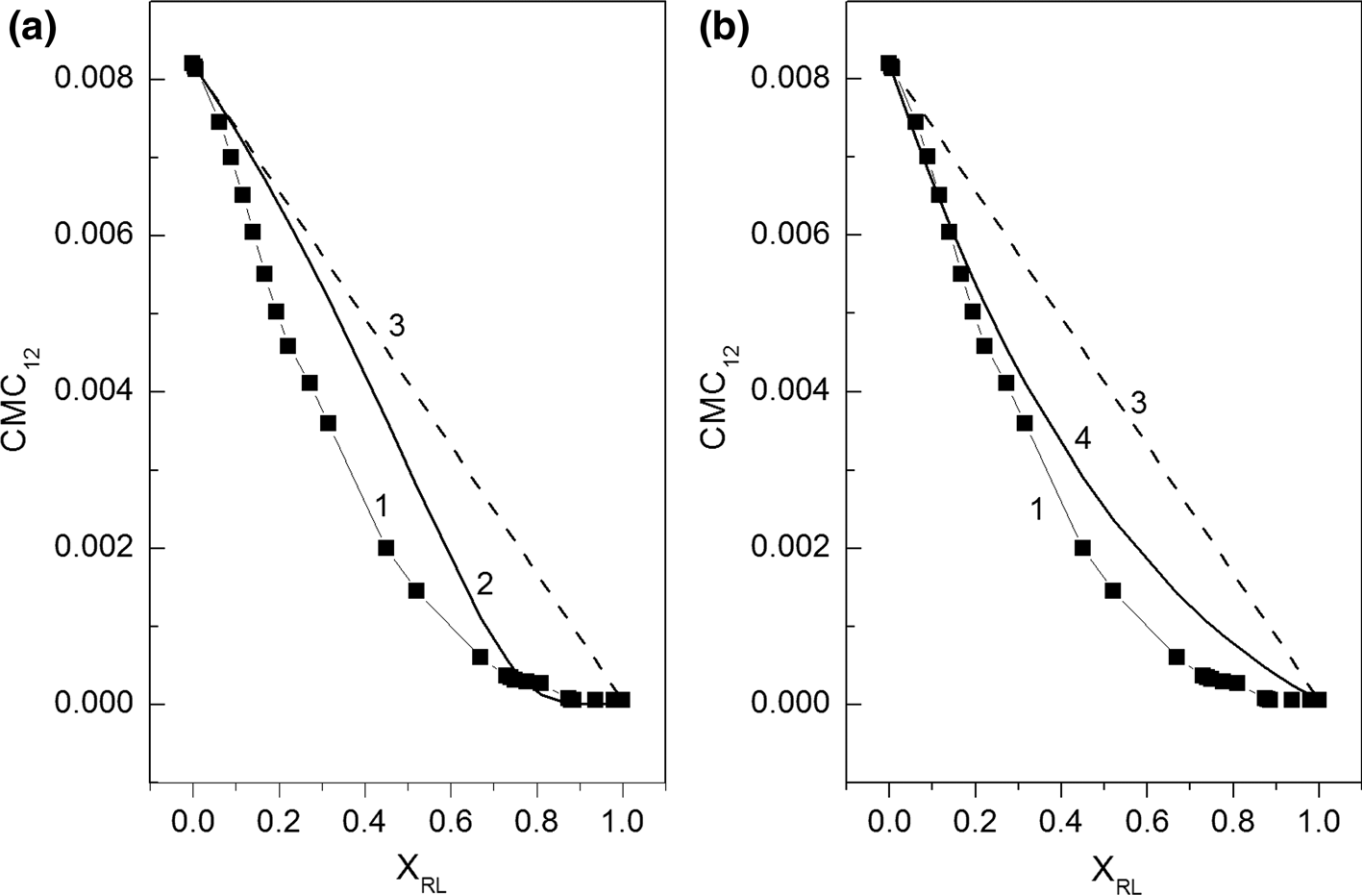
A plot of the critical micelle concentration of the RL and SDS mixture (CMC_12_) determined from the surface tension isotherm of its aqueous solution (curves 1) and *CMC*_12_calculated from Eq. (5) (Fig. 11a, curve 2), Eq. (6) (Fig. 11b, curve 4) and Eq. (4) (curves 3) *vs* the RL mole fraction in the mixed micelle (*X*_RL_)

In our case there are different head groups and hydrocarbon tails. However, we applied this equation for the calculation of the CMC of the monovalent SDS and RL anionic surfactants mixture for synergetic effect analysis. Bergström and Eriksson [3] stated that the synergetic effect depends only on CMC_2_/CMC_1_ but not on the absolute values of CMC_1_ and CMC_2_. Thus, for our calculation *λ* = CMC_2_/CMC_1_ where CMC_1_ = CMC_RL_ and CMC_2_ = CMC_SDS_ was taken. The calculated values of CMC of RL and SDS mixture from Eq. (5) are presented in Fig. 11a as a function of *X*_RL_. From Fig. 11a, the synergistic effect is most pronounced when the mixture is rich in the surfactant with a lower value of the CMC. In our case this is rhamnolipid. It is in accordance with the conclusion drawn from the Rosen *et al*. theory but only in the case of *X*_RL_ higher than 0.8.

It should be mentioned that Eq. (5) is not derived for the mixture like the one used by us. In our mixture it is difficult to establish a mutual influence on RL and SDS behaviour because of the great difference between their CMC. Therefore, we also calculated the CMC for the RL and SDS mixture by using the equation derived by Bergström and Eriksson [3] for the ionic and nonionic surfactants mixture, which was used for calculation in the form:

6 $${\text{CMC}}_{12}^{{}} \;(X_{\text{RL}} ) = (X_{\text{RL}}^{{}} )^{2} \;\exp \left( {1 - X_{\text{RL}}^{{}} } \right)\;{\text{CMC}}_{\text{RL}} + \left( {1 - X_{\text{RL}} } \right)\;\exp \left( { - X_{\text{RL}} } \right)\;{\text{CMC}}_{\text{SDS}}$$

The calculated values of CMC mixture are presented in Fig. 11b. It is interesting that in the range of *X*_RL_ from 0 to 0.2 at which the synergism was proved by Rosen *et al*. theory, the calculated values of CMC from Eq. (6) are almost the same as those determined from the isotherm of the surface tension. On the other hand, in the range of *X*_RL_ from 0.8 to 1, the values of CMC calculated from Eq. (5) are close to those obtained from the isotherm of surface tension and correspond to the synergetic effect deduced from the Rosen *et al*. theory [1, 21, 22].

**Fig. 12:**
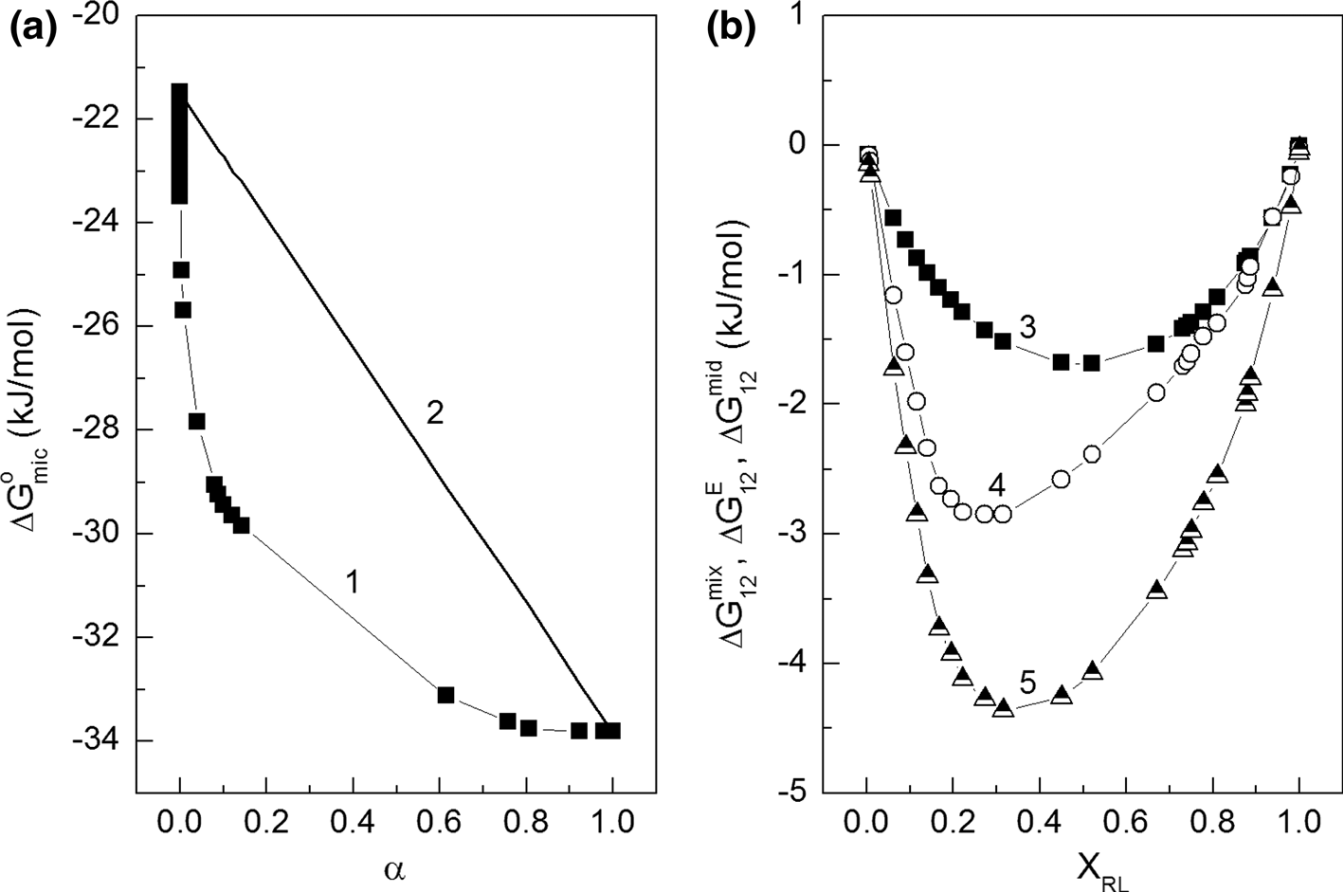
A plot of standard Gibbs free energy of micellization (Δ*G*_mic_^o^) calculated from Eq. S18 (curve 1; line 2 represents Δ*G*_mic_^o^ for ideal mixed micelles formation) *vs* the RL mole fraction in the bulk phase (*α*) (Fig. 12a) and a plot of Gibbs free energy of ideal mixing of RL and SDS in the mixed micelles (Δ*G*_12_^mid^) (curve 3), excess Gibbs free energy of nonideal mixing (Δ*G*_12_^E^) (curve 4) and Gibbs free energy of nonideal mixing of RL and SDS in the mixed micelles (Δ*G*_12_^mix^) (curve 5) calculated from Eqs. S20, S21 and S19, respectively *vs* the RL mole fraction in the mixed micelles with SDS (*X*_RL_) (Fig. 12b)

The tendency towards micelle formation of the surfactants and their mixtures is reflected in the standard Gibbs free energy of micellization (Δ*G*_mic_^o^). The changes of the values of Δ*G*_mic_^o^ calculated from Eq. S18 (Fig. 12a, curve 1) as the function of *α* are significantly different from the straight linear dependence between Δ*G*_mic_^o^ and *α* for the ideal mixed micelles. This confirms that there is nonideal mixing of SDS and RL in the micelle. It is more evident form comparison of the Gibbs free energy of mixing (Fig. 12b, curve 5; Eq. S19) to the ideal mixing of SDS and RL in the micelle (Fig. 12b, curves 3; Eq. S20). As can be seen there is significant excess of Gibbs free energy of mixing (Fig. 12b, curve 2; Eq. S21) and its minimum corresponds to the value of *X*_RL_ equal to 0.3. The negative values of the excess of Gibbs free energy of mixing indicate that the interactions between the SDS and RL molecules are stronger than between SDS–SDS and RL–RL.

### Apparent and Partial Molar Volumes of RL and SDS in Their Mixture

On the basis of the density measurements (Fig. 7), it is possible to determine not only the values of surfactant CMC but also apparent (*φ*_*V*_) and partial ($\overline{V}_{M}$) molar volumes of their molecules [26, 27]. For calculations of these values for the studied surfactants, Eqs. S22 and S23 were used. From the obtained data it shows that the presence of SDS in solution only insignificantly influences the RL apparent molar volume and *vice versa* (Figs. S10 and S11). There is small drop in the apparent molar volume of SDS and RL isotherms in the range of their concentration at which the aggregation process of the mixture takes place (Figs. S10a and S10b). There is no mutual influence of RL and SDS on their partial molar volume (Figs. S11a and S11b). The isotherms of partial molar volume of RL and SDS are different from those of apparent ones. The partial molar volumes of RL and SDS in the range of their concentrations corresponding to those in which individual surfactants are present in the monomeric form in the solution are constants. At the concentration of surfactant close or higher than their CMC, a significant increase of their partial molar volumes is observed.

Explanation of the changes of the apparent and partial molar volumes of RL and SDS was achieved on the basis of the size of their molecules and the average distance between the surfactant molecules and water in solution and between the surfactant molecules in the micelles. For this purpose it was assumed that the average distance between the surfactant hydrophilic group being in the monomeric and aggregated forms and water is nearly the same while the average distance between the hydrophobic group of surfactant and water or between the hydrophobic group in micelles can vary. Taking into account the length of the bonds between the atoms in the RL and SDS molecules and the angle between their bonds, the volume of particular groups in the molecules of studied surfactants was assumed to be equal to the volume of cubic at proper sizes. For calculations of molar volumes of surfactant molecules it was assumed that the minimal average distance cannot be lower than 1.56 Å [28] and the maximal one is the same as in hydrocarbon media (2 Å) [17]. For RL the molar volumes calculated at both average distances are equal to 407.15 ml/mol and 469.5, respectively. For SDS these values are equal to 193.56 and 274.79 ml/mol, respectively. The values of RL and SDS molar volumes calculated theoretically indicate that the changes of partial and apparent molar volumes of SDS and RL are in the range of volumes determined for the minimal and maximal average distances between the hydrocarbon part and water and between the hydrocarbon parts of surfactants.

## Conclusions

On the basis of the data obtained from the measurements of surface tension, density and viscosity of aqueous solution of SDS and RL mixtures and their discussion the following can be stated.

In the concentration range of one surfactant in the mixture corresponding to the unsaturated monolayer at the water–air interface in the absence of another, there is independent adsorption of mixture components.

If the concentration of both surfactants corresponds to their individual saturated surface layer at the water–air interface, then RL replaces SDS molecules in the mixed layer.

The mole fraction of the surfactants in the saturated mixed monolayer at the water–air interface determined on the basis of SDS and RL Gibbs surface excess concentration is nearly the same as that determined from the Hua, Rosen and Rubingh theory [1, 21, 22].

The standard Gibbs free energy of the mixture can be predicted on the basis of the Gibbs standard free energy of adsorption of individual surfactants, the mole fraction of surfactants in the mixed monolayer and the Gibbs free energy of surfactants mixing in the monolayer.

There is synergism in the reduction of the water surface tension and the micelle formation by the SDS and RL mixture.

The synergism in the mixed micelle formation was proved by the Hua, Rosen and Rubingh [1, 21, 22] as well as Bergström and Eriksson [3] theories.

The ratio of the standard Gibbs free energy of adsorption and micellization of RL to SDS, is close to that of the number of water molecules in the contact with the surfactant ones.

The changes of apparent and partial mole volume of the surfactants in their mixture can be predicted on the basis of the molecules size and average distance between the surfactant molecules in the monomeric and aggregated forms.

## Electronic supplementary material

Below is the link to the electronic supplementary material.

Supplementary material 1 (DOC 2328 kb)
