# Supplementary material for: Adsorption and Aggregation Activity of Sodium Dodecyl Sulfate and Rhamnolipid Mixture
Source: J Surfactants Deterg. 2016 Dec 10;20(2):411–23. doi: 10.1007/s11743-016-1916-6 (PMC5320015; doi:10.1007/s11743-016-1916-6)
Supplement: Supplementary file 1 — Supplementary material 1 (DOC 2328 kb) [file 11743_2016_1916_MOESM1_ESM.doc]

**ADSORPTION AND AGGREGATION ACTIVITY OF SODIUM DODECYLSULFATE AND RHAMNOLIPID MIXTURE**

DIANA MAŃKO, ANNA ZDZIENNICKA*, AND BRONISŁAW JAŃCZUK

*Department of Interfacial Phenomena, Faculty of Chemistry, Maria Curie-Skłodowska University, Maria Curie-Skłodowska Sq. 3, 20-031 Lublin, Poland*

Running title: Surface and volumetric properties

*To whom correspondence should be addressed

phone (48-81) 537-56-70

fax (48-81) 533-3348

e-mail [*aniaz@hektor.umcs.lublin.pl*](mailto:bronislaw.janczuk@poczta.umcs.lublin.pl)

**Determination of the Gibbs surface excess concentration.**

Assuming that molecules of SDS and RL are quite dissociated in the monolayer, the Gibbs surface excess concentration of SDS ($\Gamma_{SDS}$) and RL ($\Gamma_{RL}$) at the solution-air interface as well as the surfactant mixture ($\Gamma_{12}$) were determined from following equations [1,2]:

$\Gamma_{SDS}=-\frac{C_{SDS}}{2RT}\left(\frac{d\gamma_{LV}}{dC_{SDS}}\right)_{T,C_{RL}}=-\frac{1}{4.606RT}\left(\frac{d\gamma_{LV}}{d\log C_{SDS}}\right)_{T,C_{RL}}$ (S1a)

$\Gamma_{RL}=-\frac{C_{RL}}{2RT}\left(\frac{d\gamma_{LV}}{dC_{RL}}\right)_{T,C_{SDS}}=-\frac{1}{4.606RT}\left(\frac{d\gamma_{LV}}{d\log C_{RL}}\right)_{T,C_{SDS}}$ (S1b)

and

$\Gamma_{12}=-\frac{C_{12}}{2RT}\left(\frac{d\gamma_{LV}}{dC_{12}}\right)_{T}=-\frac{1}{4.606RT}\left(\frac{d\gamma_{LV}}{d\log C_{12}}\right)_{T}$ (S1c)

It appeared that in the range of low concentration of SDS ($C_{SDS}$) at constant RL one ($C_{RL}$) and vice versa as well as RL and SDS mixture ($C_{12}$) the changes of surface tension ($\gamma_{LV}$) of the aqueous solution of SDS, RL and their mixture as a function of $C_{SDS}$, $C_{RL}$ or $C_{12}$ can be expressed by the first order exponential function which allows to determine $\frac{d\gamma_{LV}}{dC_{SDS}}$, $\frac{d\gamma_{LV}}{dC_{RL}}$ and $\frac{d\gamma_{LV}}{dC_{12}}$. Introducing these values, as well as $C_{SDS}$, $C_{RL}$and $C_{12}$ into Eqs. (S1a, S1b and S1c), we calculated the values of Gibbs surface excess concentration of RL, SDS and their mixture in the range of their concentration in the bulk phase corresponding to the unsaturated monolayer of a given surfactant and SDS and RL mixture. The maximal Gibbs surface excess concentration of SDS ($\Gamma_{SDS}^{\max}$), RL ($\Gamma_{RL}^{\max}$) and the mixture ($\Gamma_{12}^{\max}$) corresponding to the saturated monolayer at the water-air interface were determined from the linear relation between $\gamma_{LV}$ and $\log C_{SDS}$ or $\log C_{RL}$ or $\log C_{12}$.

**Calculation of the surface tension of aqueous solution of SDS and RL mixture on the basis of surface tension of the individual surfactants solution**

If it is possible to assume that the components of aqueous solutions of surfactant mixture are adsorbed independently at the water-air interface, than the surface tension of solution can be predicted on the basis of the following equation:

$\gamma_{LV}=\gamma_{W}-\pi_{SDS}-\pi_{RL}$ (S2)

where $\gamma_{LV}$ is the surface tension of aqueous solution of RL and SDS mixture,$\gamma_{W}$ is the water surface tension, $\pi_{SDS}$ and $\pi_{RL}$ are respectively the differences between the water and aqueous solution of SDS or RL surface tension, respectively corresponding to the concentration of these surfactants in the aqueous solutions of their mixture.

In the case of the ideal mixture of homologue surfactants Fainermann and Miller [3,4] for calculation of surface tension of the aqueous solution of their mixture on the basis of surface tension of individual surfactants proposed the equation of state in the form:

$\exp\overline{\Pi}=\exp\overline{\Pi}_{1}+\exp\overline{\Pi}_{2}-1$ (S3)

where $\overline{\Pi}=\Pi\omega/RT$, $\overline{\Pi}_{1}=\Pi{}_{1}\omega_{1}/RT$, $\overline{\Pi}_{2}=\Pi_{2}\omega_{2}/RT$are the dimensionless surface pressures of the mixture and individual solutions of surfactants 1 and 2, respectively, *ω­*_1_, *ω­*_2_ and *ω­* is the molar surface area of the surfactants 1, 2 and their mixture, respectively. In the case of two homologues $\omega_{1}=\omega_{2}=\omega$. In the case of SDS and RL the $\omega_{SDS}$ and $\omega_{RL}$are equal to 2.1 x 10^5^ and 4.16 x 10^5^ m^2^/mol, respectively. The surface pressures of SDS and RL are taken from the literature [5,6].

**Determination of the area occupied by the surfactants at the water-air interface and composition of the mixed monolayer**

To determine the area occupied by SDS and RL molecules at the water-air interface the water surface excess concentration ($\Gamma_{o}$) must be calculated using the following equation [7]:

$\Gamma_{o}NA_{o}+\Gamma_{SDS}NA_{SDS}+\Gamma_{RL}NA_{RL}=1$ (S4)

where: *N* is the Avogadro number, *A*_o_, $A_{SDS}$ and $A_{RL}$ are the minimal surface areas per molecule of water (10 Ǻ^2^), SDS (35 Ǻ^2^) [5] and RL (69.08 Ǻ^2^) [6], respectively.

Knowing $\Gamma_{o}$, $\Gamma_{SDS}$ and $\Gamma_{RL}$, it is possible to establish the area occupied by SDS ($X_{SDS}^{S}$) and RL ($X_{RL}^{S}$) molecules at the water-air interface from the following equations:

$X_{SDS}^{S}=\frac{\Gamma_{SDS}}{\Gamma_{o}+\Gamma_{SDS}+\Gamma_{RL}}$ (S5)

$X_{RL}^{S}=\frac{\Gamma_{RL}}{\Gamma_{o}+\Gamma_{SDS}+\Gamma_{RL}}$ (S6)

In turn, the mole fraction of SDS ($X_{SDS}$) and RL ($X_{RL}$) in their mixed monolayer can be calculated using the equations:

$X_{SDS}=\frac{\Gamma_{SDS}}{\Gamma_{SDS}+\Gamma_{RL}}$ (S7)

$X_{RL}=1-X_{SDS}$ (S8)

The mole fraction of SDS and RL in the mixed monolayer can be also determined on the basis of the Rubingh and Rosen theory [1,8,9]. They derived the equation for calculation of the composition of mixed monolayer, which for SDS and RL mixture can be written in the form:

$\frac{\left(X_{SDS}\right)^{2}\ln\left(\alpha C_{12}/X_{SDS}C_{SDS}^{o}\right)}{\left(1-X_{SDS}\right)^{2}\ln\left[\left(1-\alpha\right)C_{12}/\left(1-X_{SDS}\right)C_{RL}^{o}\right]}=1$ (S9)

where $\alpha$ is the mole fraction of RL in the RL with SDS mixture in the bulk phase, $C_{SDS}^{o}$, $C_{RL}^{o}$ and $C_{12}$ are the molar concentrations in the bulk phase of SDS, RL and their mixture, respectively, required to produce a given surface tension value.

If we know the mole fraction of components in the mixed monolayer, then applying the Rubingh and Rosen equation [1,8,9] it is possible to determine the parameter of intermolecular interactions of surfactants molecules ($\beta^{\delta}$) in the mixed monolayer which, in turn, allows to consider the presence of synergetic effect in the reduction of water surface tension by this monolayer. The Rubingh and Rosen equation has the form [1,8,9]:

$\beta^{\delta}=\frac{\ln(\alpha C_{12}/X_{SDS}C_{SDS}^{0})}{(1-X_{SDS})^{2}}$ (S10)

On the basis of $\beta^{\delta}$, $X_{SDS}$ and $X_{RL}$, it is possible to calculate the activity coefficients ($f$) of SDS and RL in the mixed monolayer using the following equations [1,8,9]:

$\ln f_{RL}=\beta^{\delta}\left(1-X_{RL}\right)^{2}$ (S11a)

and

$\ln f_{SDS}=\beta^{\delta}\left(X_{RL}\right)^{2}$ (S11b)

**Determination of the standard Gibbs free energy of adsorption**

The Gibbs standard free energy of adsorption ($\Delta G_{ads}^{o}$) can be determined using different methods among which the Langmuir equation modified by de Boer is commonly used [10]:

$\frac{A_{0}}{A-A_{0}}\exp\frac{A_{0}}{A-A_{0}}=\frac{C}{\omega}\exp\left(\frac{-\Delta G_{ads}^{0}}{RT}\right)$ (S12)

where *A* is the area occupied per molecule at the water-air interface, *A*_o_ is the “excluded area”, i.e., the area of the interface inaccessible to one molecule due to the presence of another

one and $\omega$ is the number of water molecules in dm^3^.

The values of $A_{0}$ for SDS and RL mixture were calculated using the limiting area of individual surfactants and their mole fraction in the mixed monolayer.

For determination of $\Delta G_{ads}^{o}$for SDS and RL mixture, there were also applied the following equations:

$\Delta G_{ads}^{o}=\alpha G_{ads}^{o}(RL)+(1-\alpha)\Delta G_{ads}^{o}(SDS)$ (S13a)

$\Delta G_{ads}^{o}=X_{RL}G_{ads}^{o}(RL)+(1-X_{RL})\Delta G_{ads}^{o}(SDS)$ (S13b)

and

$\Delta G_{ads}^{o}=X_{RL}G_{ads}^{o}(RL)+(1-X_{RL})\Delta G_{ads}^{o}(SDS)+RT(X_{RL}\ln f_{RL}+X_{SDS}\ln f_{SDS})$ (S13c)

where $G_{ads}^{o}(RL)$ and $G_{ads}^{o}(SDS)$ are the Gibbs standard free energy of adsorption of RL in absence of SDS and vice versa, respectively.

**Determination of the mixed micelle CMC**

The values of CMC for the ideal mixture of two surfactants ($CMC_{12}$) can be determined from the equation, which for the SDS and RL mixture can be written in the form [1]:

$\frac{1}{CMC_{12}}=\frac{1-\alpha}{CMC_{SDS}}+\frac{\alpha}{CMC_{RL}}$ (S14)

where $CMC_{SDS}$and $CMC_{RL}$ are the CMC of individual surfactants, $\alpha$ and $1-\alpha$ are the mole fractions of individual surfactants in the mixture in the bulk phase.

**Composition of the mixed micelle and standard Gibbs free energy of micellization**

According to the Rubingh and Rosen theory [1,8,9], the mole fraction of TX-100 and RL can be determined from the following equation:

$\frac{\left(X_{SDS}^{M}\right)^{2}\ln\left(\alpha CMC_{12}/X_{SDS}CMC_{SDS}\right)}{\left(1-X_{SDS}^{M}\right)^{2}\ln\left[\left(1-\alpha\right)CMC_{12}/\left(1-X_{SDS}^{M}\right)CMC_{RL}\right]}=1$ (S15)

where $CMC_{SDS}$, $CMC_{RL}$, $CMC_{12}$ are the critical micelle concentrations (CMC) of the individual SDS, RL and their mixture, respectively, and $X_{SDS}^{M}$is the mole fraction of SDS in the mixed micelle.

On the basis of the mole fraction of SDS or RL in the bulk phase, CMC of SDS or RL and mixture as well as the mole fraction of SDS or RL in the mixed micelle, it is possible to determine the parameter of intermolecular interactions *β^M^*, from the Rubingh and Rosen relation which for our system has the form [1,8,9]:

$\beta^{M}=\frac{\ln(\alpha C_{12}^{M}/X_{SDS}^{M}C_{SDS}^{M})}{(1-X_{SDS}^{M})^{2}}$ (S16)

Using $\beta^{M}$, $X_{SDS}^{M}$ and $X_{RL}^{M}$, the activity coefficients of SDS ($f_{SDS}^{M}$) and RL ($f_{RL}^{M}$) can be established from the following equations:

$\ln f_{SDS}^{M}=\beta^{M}\left(1-X_{SDS}^{M}\right)^{2}$ (S17a)

and

$\ln f_{RL}^{M}=\beta^{M}\left(X_{SDS}^{M}\right)^{2}$ (S17b)

In turn, on the basis of *β^M^*, it is possible to deduce the possibility of the synergetic effect in the mixed micelle formation.

The standard Gibbs free energy of micellization of SDS and RL mixture can be estimated from equation [1]:

$\Delta G_{mic(12)}^{o}=RT\ln\frac{CMC}{\omega}$ (S18)

The standard Gibbs free energy of SDS and RL mixture micellization ($\Delta G_{mic(12)}^{o}$) was also determined from the following equations:

$\begin{matrix} \Delta G_{12}^{mix}=RT\left[X_{RL}\ln X_{RL}+(1-X_{RL})\ln(1-X_{RL})+X_{RL}\ln f_{RL}+(1-X_{RL})\ln f_{SDS}\right]= \\ \Delta G_{12}^{mid}+\Delta G_{12}^{E} \end{matrix}$ (S19)

where

$\Delta G_{12}^{mid}=RT[X_{RL}\ln X_{RL}+(1-X_{RL})\ln(1-X_{RL})]$ (S20)

and

$\Delta G_{12}^{E}=RT(X_{RL}\ln f_{RL}+(1-X_{RL})\ln f_{SDS})$ (S21)

**Apparent and partial molar volumes of surfactants**

The mixed micelle formation should be reflected by the changes of the apparent ($\varphi_{V}$) and partial molar volumes ($\overline{V}_{M}$) which can be determined on the basis of the solution density from the following equations [11,12]:

$\varphi_{V}=\frac{M_{S}}{\rho_{0}}+\frac{1000\left(\rho_{0}-\rho\right)}{C_{S}\rho_{0}}$ (S22)

$\overline{V}_{M}=\frac{M_{S}}{\rho}\left[1-\frac{\left(100-C_{p}\right)}{\rho}\frac{d\rho}{dC_{p}}\right]$ (S23)

where *M*_S_ is the molecular weight of surfactant, *C*_p_ is the percentage weight of the solute, *C*_S_ is the concentration of surfactant in mol/cm^3^, $\rho$is the density of the solution, $\rho_{0}$ is the density of the aqueous solution of SDS or RL.

To determine $\overline{V}_{M}$, it is necessary to know the dependence between density and *C*_p_. For all studied solutions at both $C_{SDS}$ and $C_{RL}$ constants, this dependence can be fitted by the following polynomial:

$\rho=c+kC_{P}+sC_{P}^{2}$ (S24)

where *c*, *k* and *s* are the constants.

The values of *c*, *k* and *s* in Eq. (S29) depend on the constant $C_{SDS}$or $C_{RL}$ in the aqueous solutions.

**References**

[1] Adamson AW, Gast AP (1997) Physical Chemistry of Surfaces, Wiley-Interscience New York.

[2] Rosen JM (2004) Surfactants and Interfacial Phenomena, Wiley Interscience New York.

[3] Fainerman VB, Miller R, Aksenenko EV (2002) Simple model for prediction of surface

tension of mixed surfactant solutions. Adv Colloid Interface Sci 96: 339–359.

# [4] Fainerman VB, Miller R (2001) Simple method to estimate surface tension of mixed surfactant solutions. J Phys Chem B 105: 11432–11438.

# [5] Zdziennicka A, Szymczyk K, Krawczyk , Jańczuk B (2012) Activity and thermodynamic parameters of some surfactants adsorption at the water-air interface. Fluid Phase Equilib 318: 25–33.

[6] Mańko D, Zdziennicka A, Jańczuk B (2014) Thermodynamic properties of rhamnolipid micellization and adsorption. Colloid Surf B 119: 22–29.

[7] Chattoraj DK, Birdi KS (1984) Adsorption and the Gibbs Surface Excess, Plenum Press New York and London.

# [8] Hua XY, Rosen MJ (1982) Calculation of the coefficient in the Gibbs equation for the adsorption of ionic surfactants from aqueous binary mixtures with nonionic surfactants. J Colloid Interface Sci 87: 469–477.

# [9] Rubingh DN (1979) Mixed Micelle Solutions. In: Mittal KL (ed) Solution Chemistry of Surfactants. Plenum New York, pp. 337–354.

[10] de Boer JH (1953) The Dynamic Character of Adsorption, Clarendon Press Oxford.

[11] Kale KM, Zana R (1977) Effect of the nature of the counterion on the volume change upon micellization of ionic detergents in aqueous solutions. J Colloid Interface Sci 61: 312–322.

[12] Benjamin L (1966) Partial molal volume changes during micellization and solution of nonionic surfactants and perfluorocarboxylates using a magnetic density balance. J Phys Chem 70: 3790–3797.


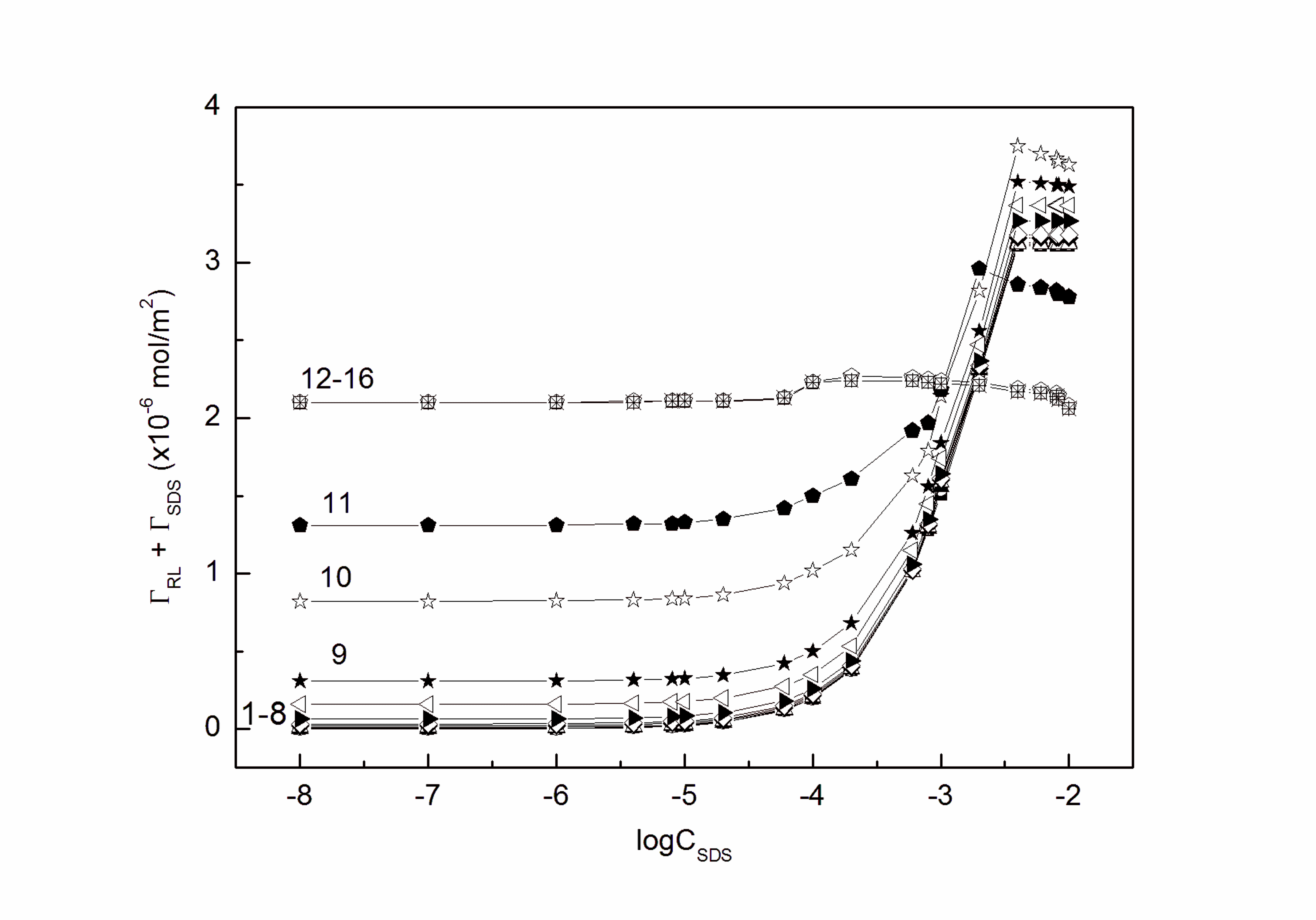


Fig. S1. A plot of the total Gibbs surface excess concentration of SDS and RL ($\Gamma_{SDS}+\Gamma_{RL}$) vs. the logarithm of SDS concentration in the bulk phase ($C_{SDS}$). Curves 1 – 16 correspond to the constant RL concentration equal to 3.97 x 10^-10^; 9.92 x 10^-10^; 2.48 x 10^-9^; 5.95 x 10^-9^; 1.24 x 10^-8^; 1.98 x 10^-8^; 3.97 x 10^-8^; 9.92 x 10^-8^; 1.98 x 10^-7^; 9.92 x 10^-7^; 1.98 x 10^-6^; 9.9 x 10^-6^; 1.98 x 10^-5^; 3.97 x 10^-5^; 6.35 x 10^-5^ and 7.94 x 10^-5^ M.


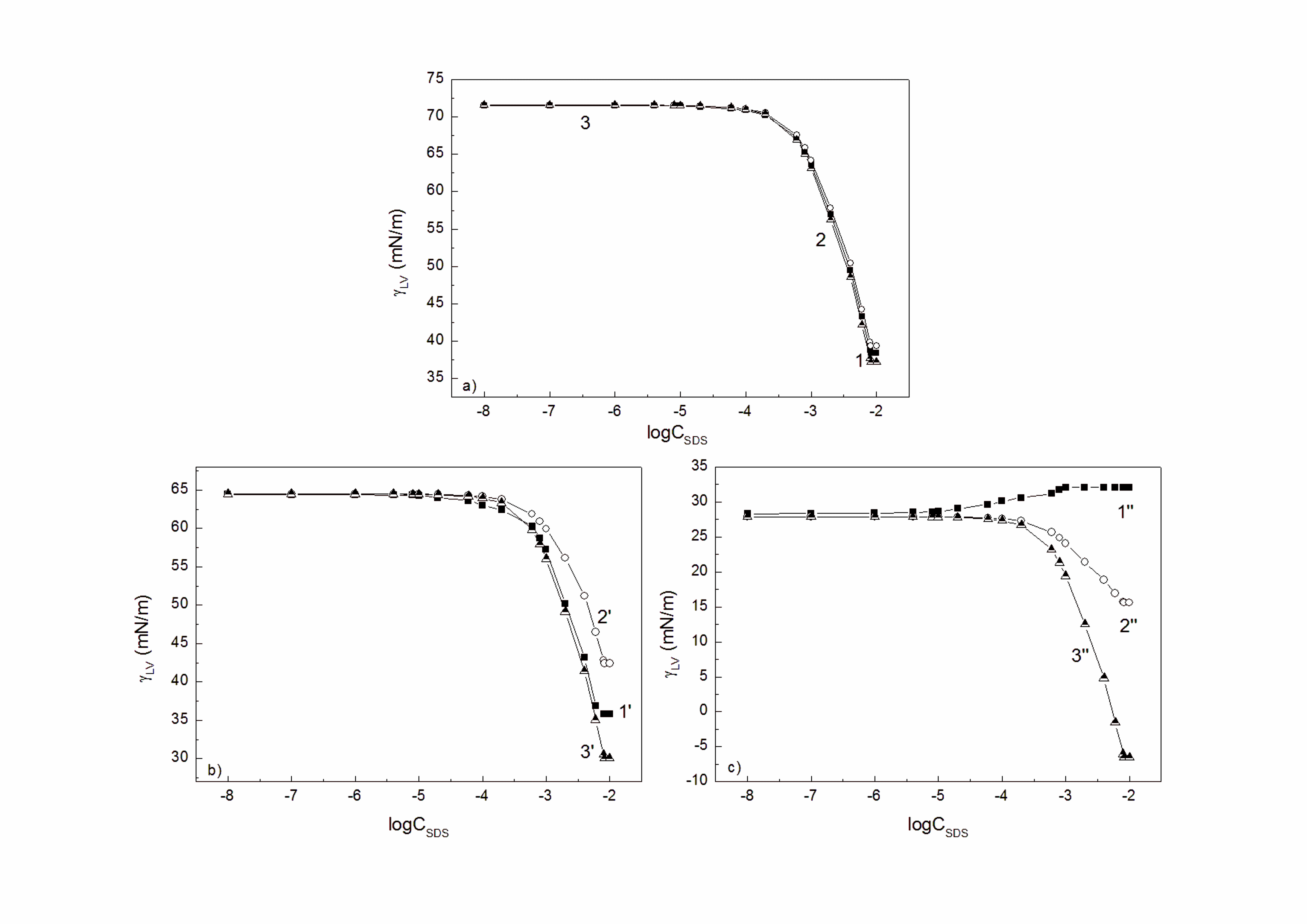


Fig. S2. A plot of the surface tension ($\gamma_{LV}$) of aqueous solution of SDS and RL mixture at the constant RL concentration equal to 0.0002 mg/dm^3^ (3.97 x 10^-10^ M) (a), 0.5 mg/dm^3^ (9.92 x 10^-7^M) (b) and 10 mg/dm^3^ (1.98 x 10^-5^M) (c) vs. the logarithm of SDS concentration in the bulk phase ($C_{SDS}$). Curves 1, 1’ and 1’’ correspond to the measured values of ($\gamma_{LV}$), curves 2, 2’, 2’’ and 3, 3’, 3’’ to those calculated from Eqs. (S2) and (S3), respectively.


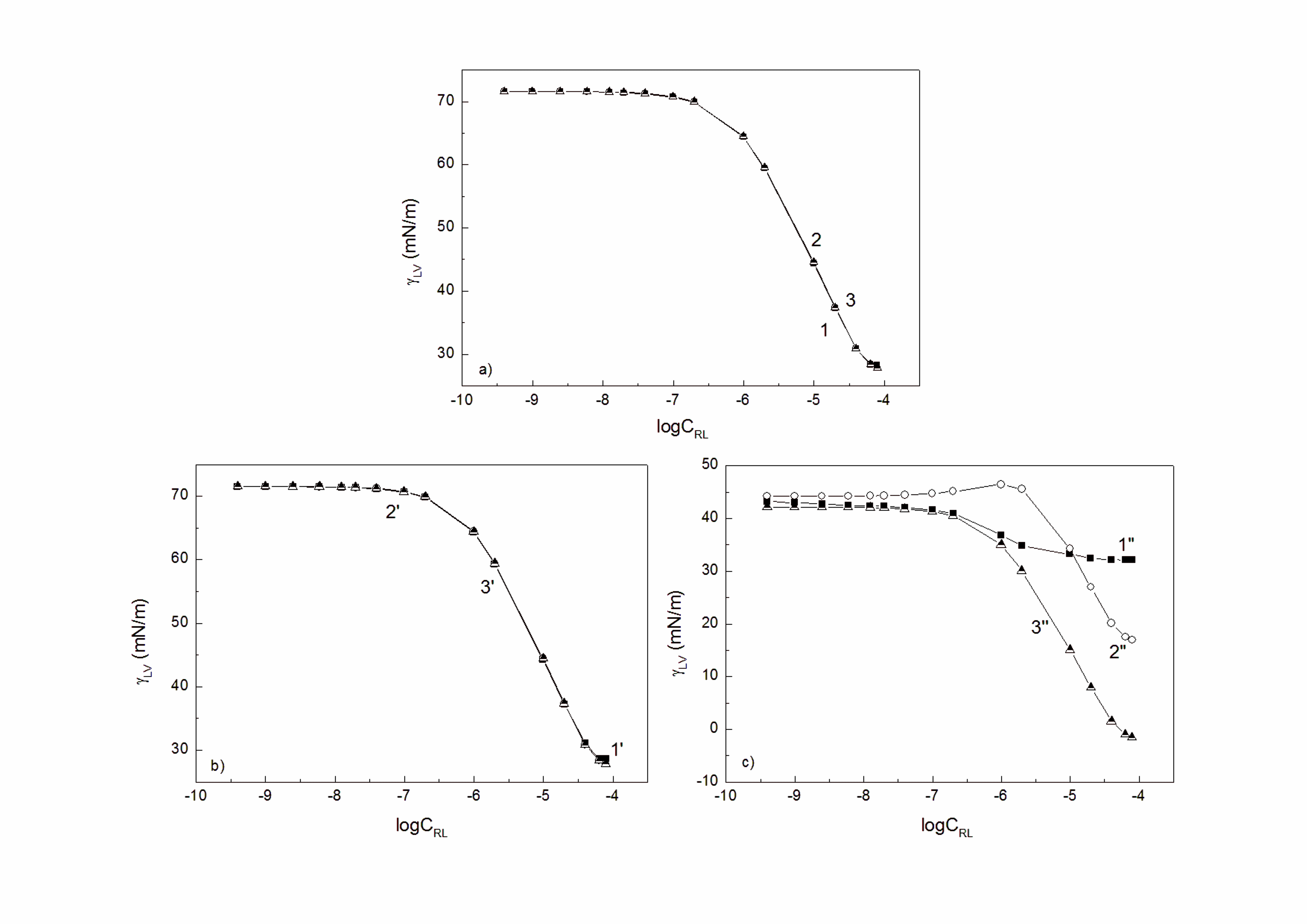


Fig. S3. A plot of the surface tension ($\gamma_{LV}$) of aqueous solution of SDS and RL mixture at the constant SDS concentration equal to 1 x 10^-8^ M (a), 1 x 10^-5^ M (b) and 6 x 10^-3^ M (c) vs. the logarithm of RL concentration in the bulk phase ($C_{RL}$). Curves 1, 1’ and 1’’ correspond to the measured values of ($\gamma_{LV}$), curves 2, 2’, 2’’ and 3, 3’, 3’’ to those calculated from Eqs. (S2) and (S3), respectively.


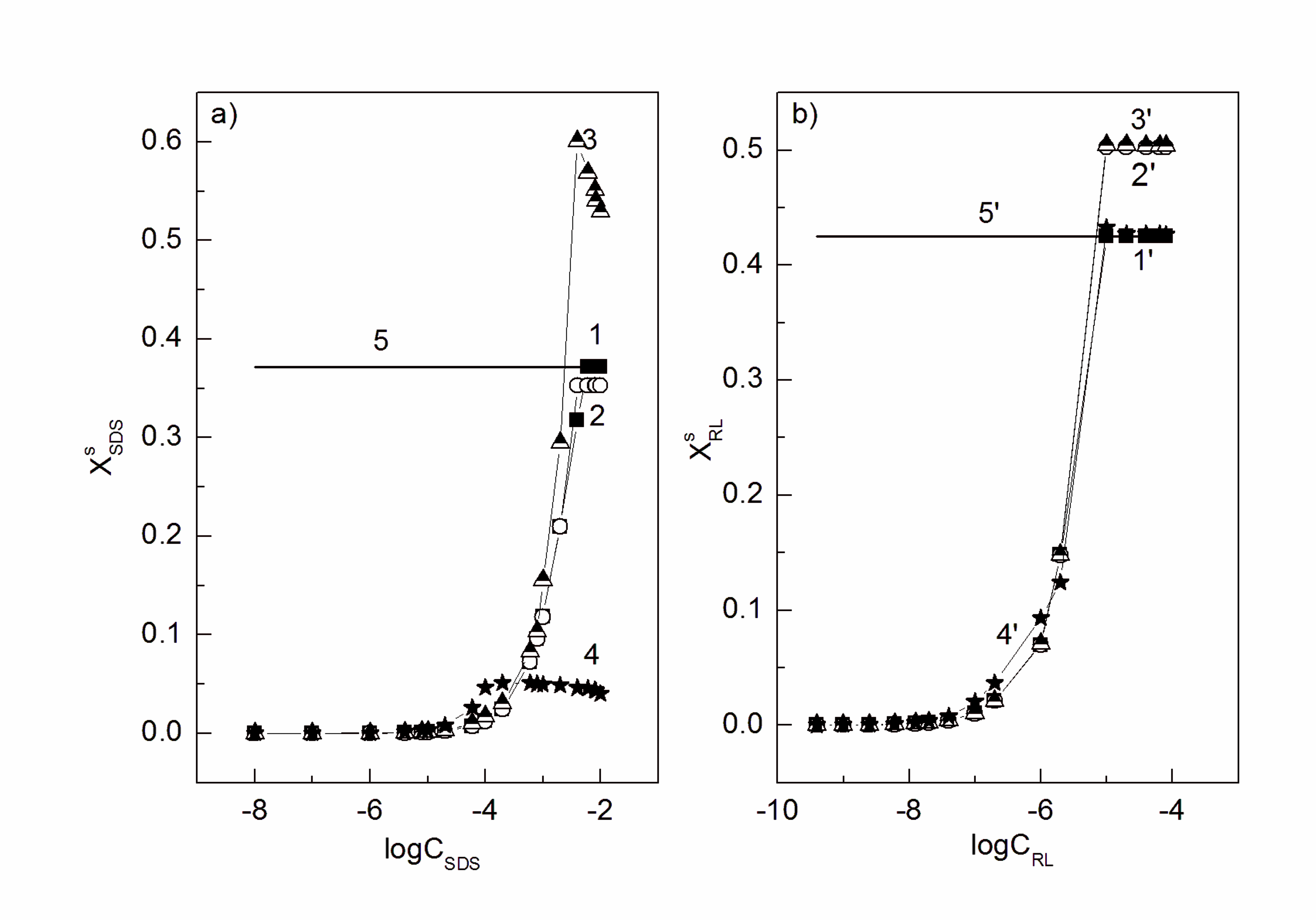


Fig. S4. A plot of the fraction of area occupied by SDS ($X_{SDS}^{S}$) (a) and RL ($X_{RL}^{S}$) molecules (b) at the water-air interface vs. the logarithm of their concentration in the bulk phase. Curve 1 corresponds to the fraction of area occupied by SDS molecules in the absence of RL, curves 2 – 4 correspond to the fraction of area occupied by SDS molecules at the constant RL concentration equal to 0.00125 mg/dm^3^ (2.48 x 10^-9^); 0.5 mg/dm^3^ (9.92 x 10^-7^M) and 10 mg/dm^3^ (1.98 x 10^-5^M), curve 1' corresponds to the fraction of area occupied by RL molecules in absence of SDS, curves 2’– 4’ correspond to the fraction of the area occupied by RL molecules at constant SDS concentration equal to 1 x 10^-8^ M, 1 x 10^-5^ M and 6 x 10^-3^ M. Lines 5 and 5’ represent the maximal fraction of area occupied by SDS molecules in the absence of RL and RL in the absence of SDS, respectively.


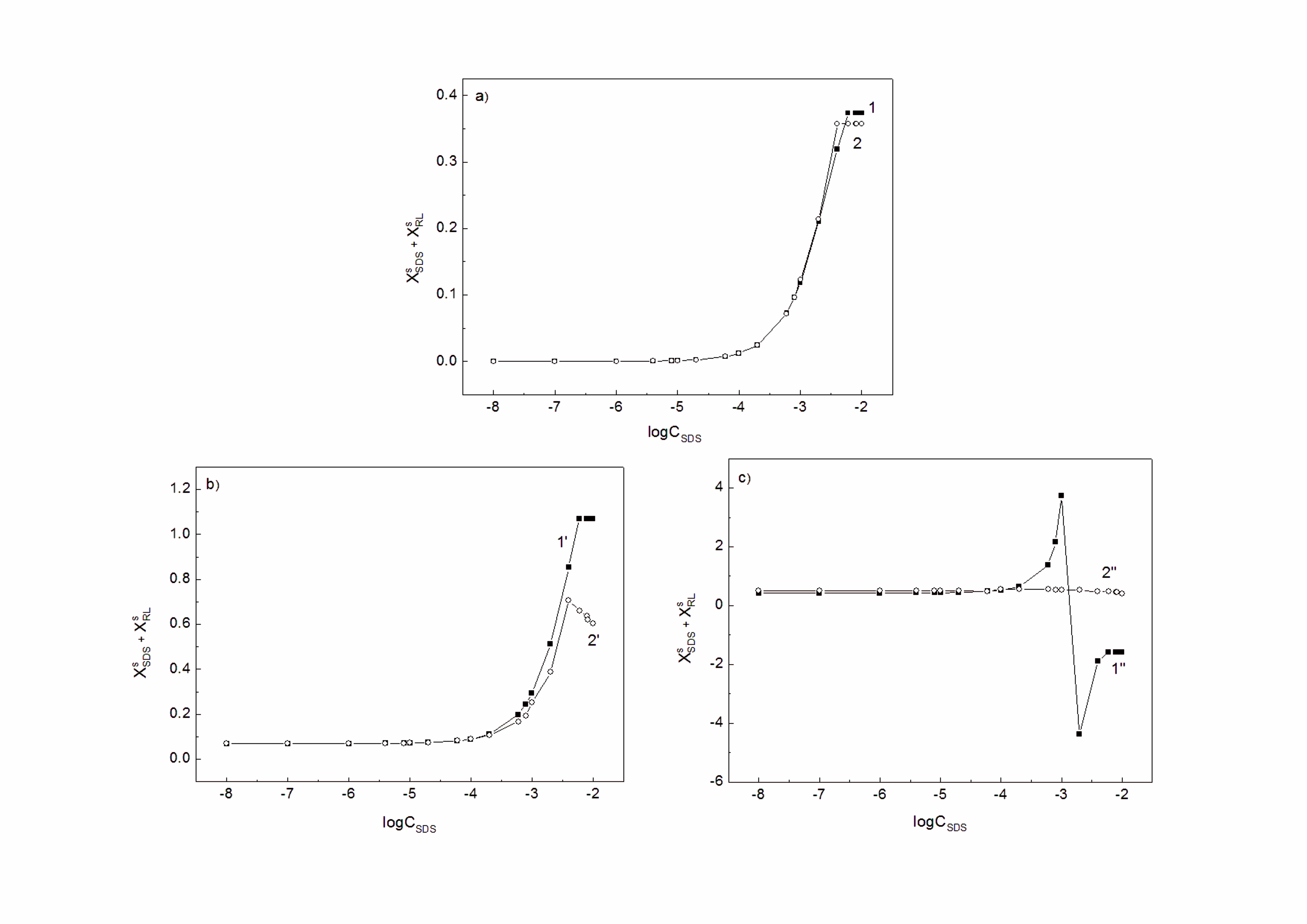


Fig. S5. A plot of the total fraction of the area occupied by SDS and RL molecules at the water-air interface $\left(X_{SDS}^{s}+X_{RL}^{s}\right)$ calculated from the independent adsorption using the surface excess concentration of individual surfactants (curves 1, 1’ and 1’’) and for the mixtures (curves 2, 2’ and 2’’) vs. the logarithm of SDS concentration ($C_{SDS}$). Curves 1 and 2 correspond to the constant RL concentration equal to 0.00125 mg/dm^3^ (2.48 x 10^-9^) (a), curves 1’ and 2’ to 0.5 mg/dm^3^ (9.92 x 10^-7^M) (b) and curves 1’’ and 2’’ to10 mg/dm^3^ (1.98 x 10^-5^M) (c).

#
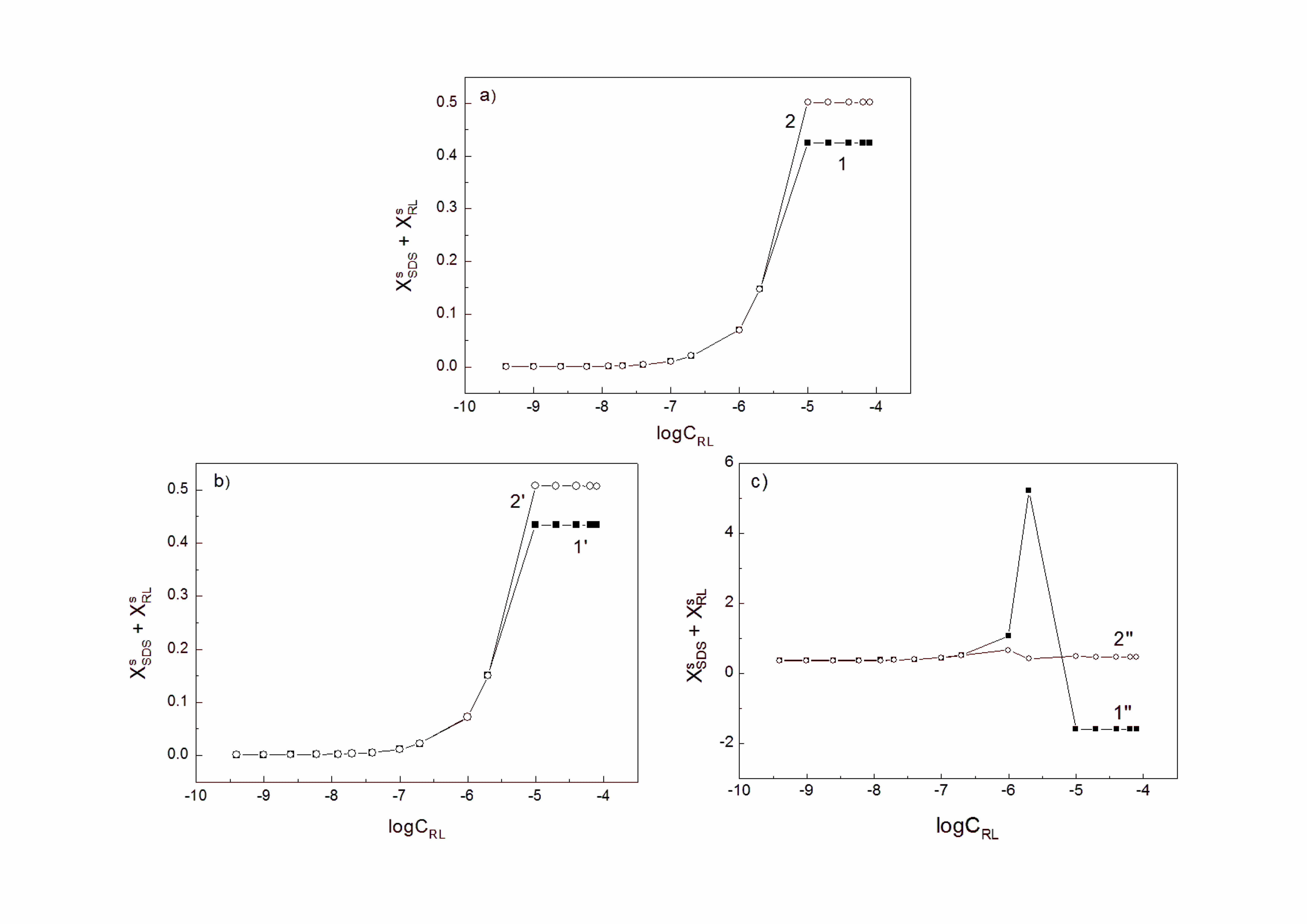


Fig. S6. A plot of the total fraction of the area occupied by SDS and RL molecules at the water-air interface $\left(X_{SDS}^{s}+X_{RL}^{s}\right)$ calculated from the independent adsorption using the surface excess concentration of individual surfactants (curves 1, 1’ and 1’’) and for the mixtures (curves 2, 2’ and 2’’) vs. the logarithm of RL concentration ($C_{RL}$). Curves 1and 2 correspond to the constant SDS concentration equal to 1 x 10^-8^ M (a), curves 1’ and 2’ to 1 x 10^-5^ M (b) and curves 1’’ and 2’’ to 6 x 10^-3^ M (c).

#
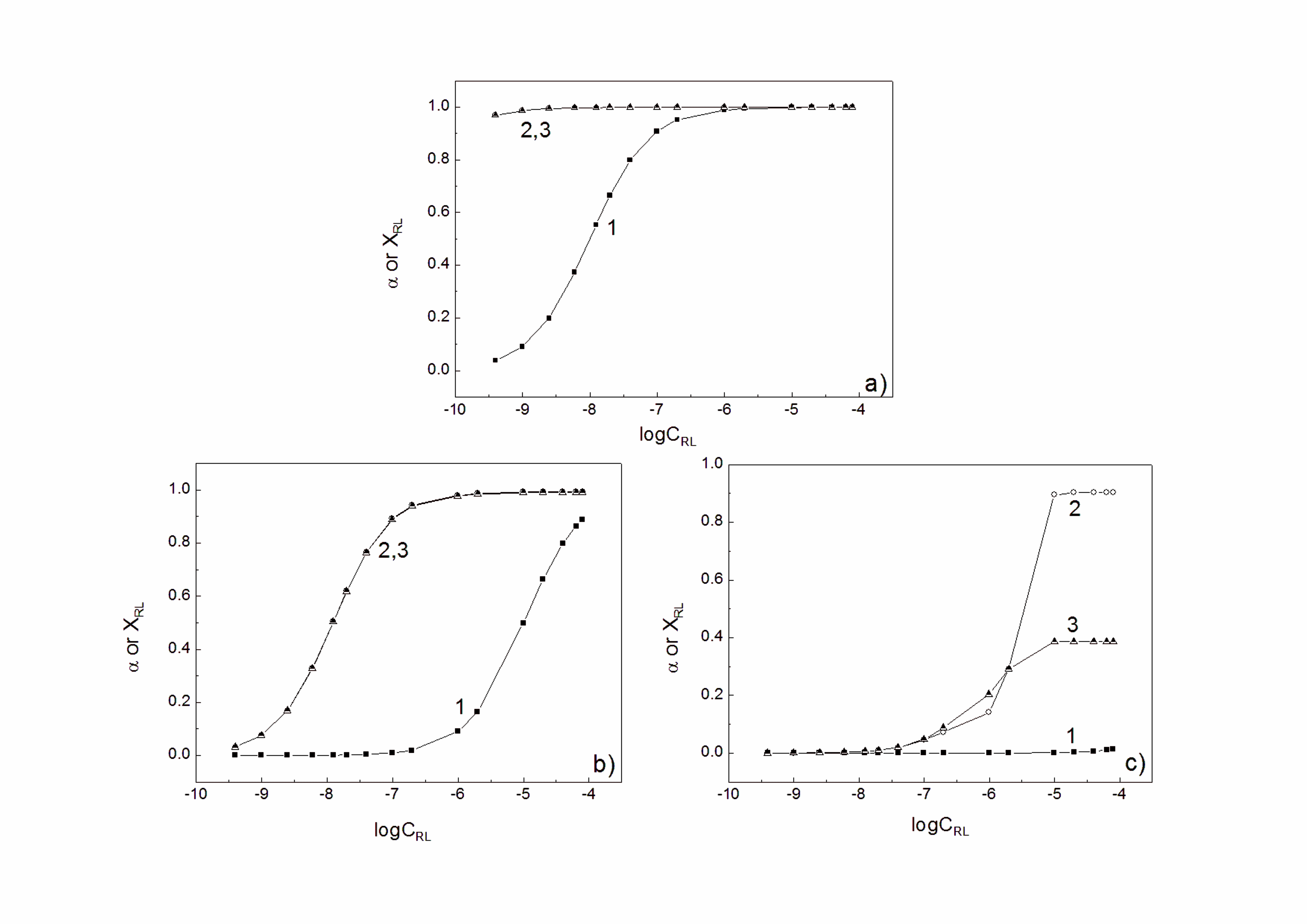


Fig. S7. A plot of the mole fraction of RL in the bulk phase (curve 1) ($\alpha$) and in the mixed monolayer ($X_{RL}$) (curves 2 and 3) at the constant SDS concentration equal to
1 x 10^-8^ M (a), 1 x 10^-5^ M (b) and 6 x 10^-3^ M (c) vs. the logarithm of RL concentration ($C_{RL}$). Curves 2 and 3 correspond to the mole fraction of RL calculated from the independent and "real" Gibbs surface excess concentration of RL and SDS.

#
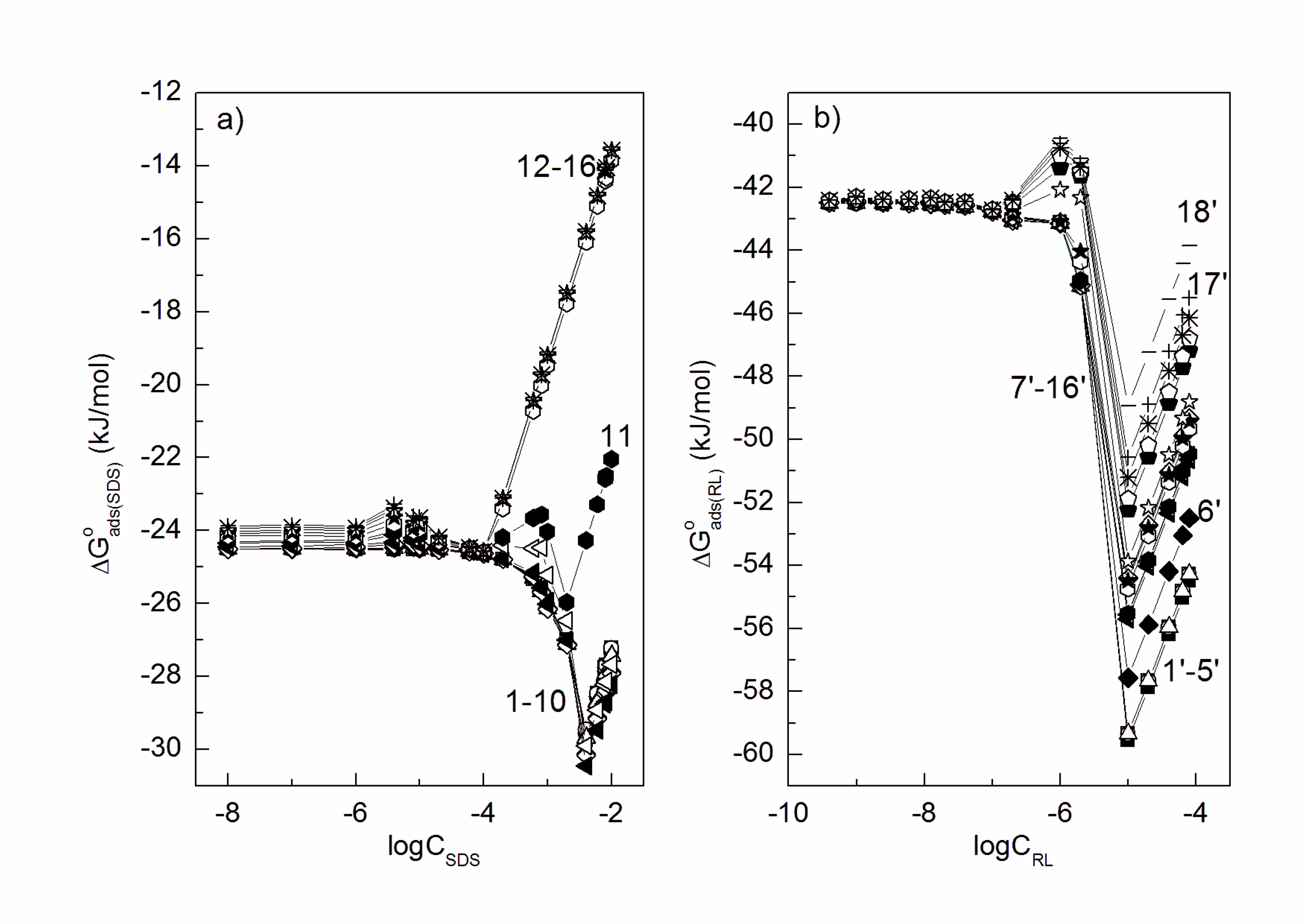


Fig. S8. A plot of the standard Gibbs free energy of SDS (a) and RL (b) adsorption ($\Delta G_{ads}^{o}$) calculated from Eq. S12 vs. the logarithm of their concentration. Curves 1 – 16 correspond to the constant RL concentration ($C_{RL}$) equal to 3.97 x 10^-10^; 9.92 x 10^-10^; 2.48 x 10^-9^; 5.95 x 10^-9^; 1.24 x 10^-8^; 1.98 x 10^-8^; 3.97 x 10^-8^; 9.92 x 10^-8^; 1.98 x 10^-7^; 9.92 x 10^-7^; 1.98 x 10^-6^; 9.9 x 10^-6^; 1.98 x 10^-5^; 3.97 x 10^-5^; 6.35 x 10^-5^ and 7.94 x 10^-5^ M. Curves 1’ – 18’ correspond to the constant SDS concentration equal to 1 x 10^-8^; 1 x 10^-7^; 1 x 10^-6^; 4 x 10^-6^; 8 x 10^-6^; 1 x 10^-5^; 2 x 10^-5^; 6 x 10^-5^; 1 x 10^-4^; 2 x 10^-4^; 6 x 10^-4^; 8 x 10^-4^; 1 x 10^-3^; 4 x 10^-3^; 6 x 10^-3^, 8 x 10^-3^, 1 x 10^-2^ and 1 x 10^-2^ M, respectively.

#
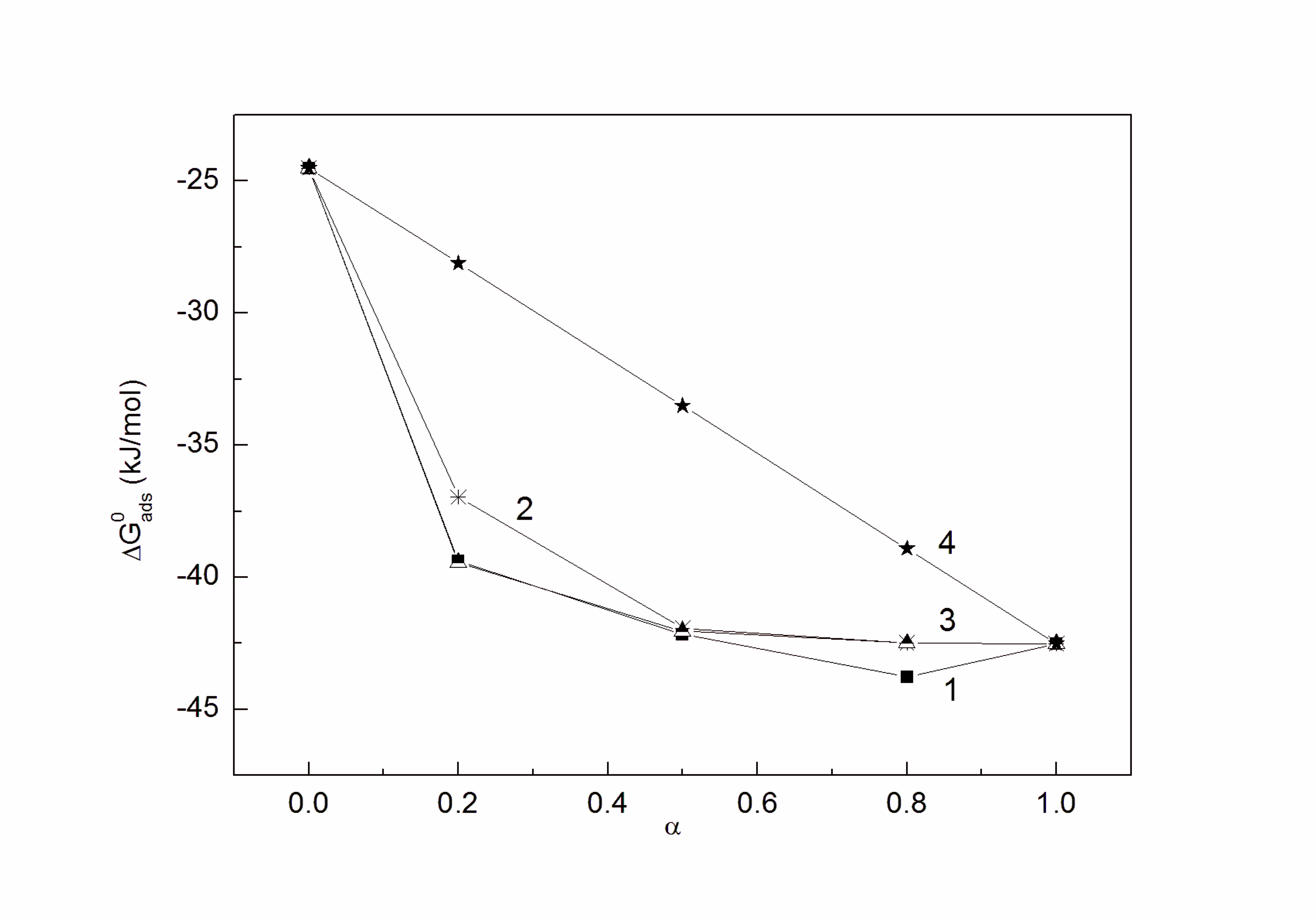


Fig. S9. A plot of standard Gibbs free energy of adsorption ($\Delta G_{ads}^{o}$) calculated from Eq. (S12) (curve 1), Eq. S13b (curve 2), Eq. (S13c) (curve 3) and Eq. (13a) (curve 4) vs. the mole fraction of RL in the mixture (α).


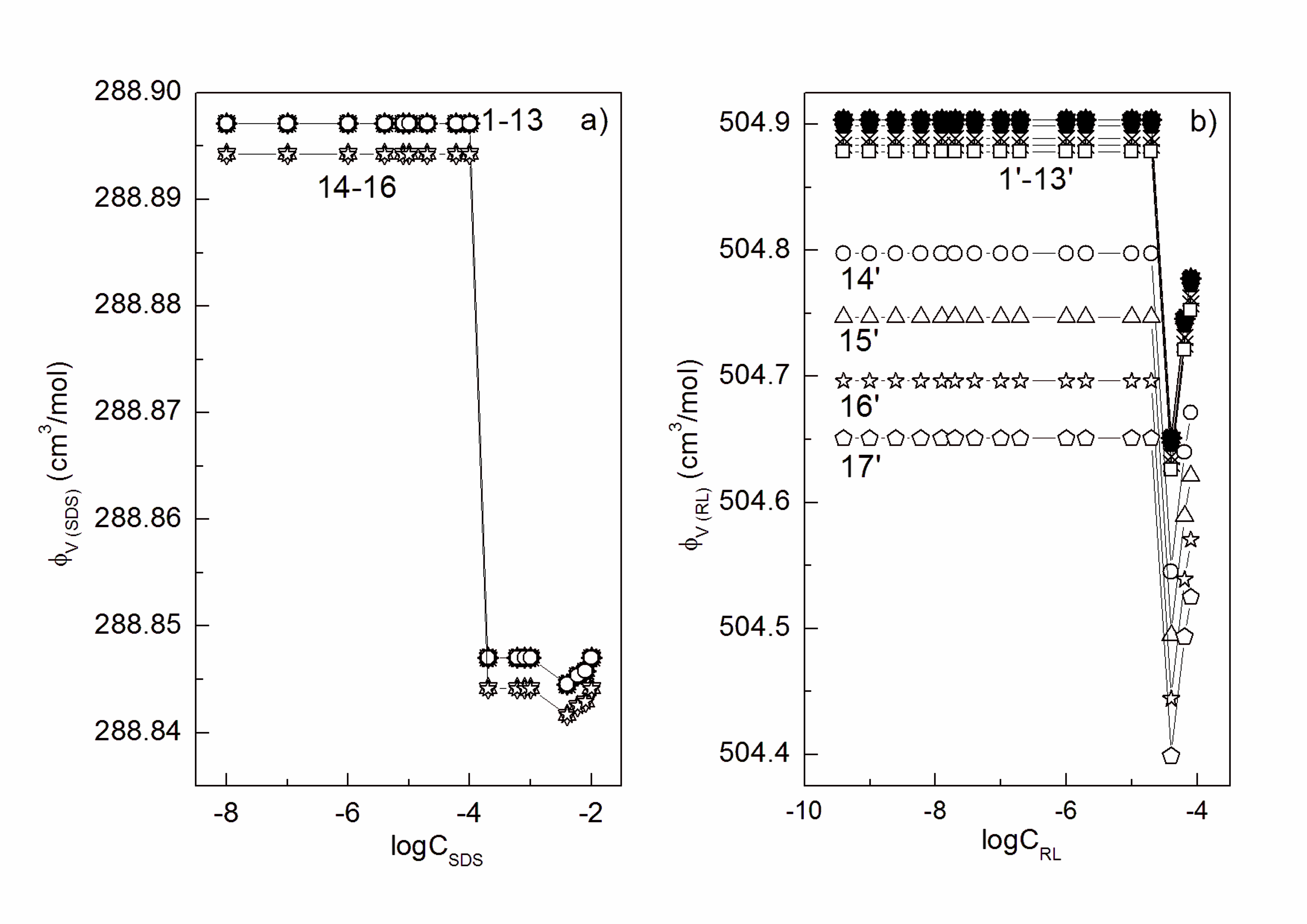


Fig. S10. A plot of the apparent molar volume ($\varphi_{V}$) of SDS (a) and RL (b) calculated from Eq. S22 vs. the logarithm of surfactant concentration. Curves 1 – 16 correspond to the constant RL concentration ($C_{RL}$) equal to 3.97 x 10^-10^; 9.92 x 10^-10^; 2.48 x 10^-9^;
5.95 x 10^-9^; 1.24 x 10^-8^; 1.98 x 10^-8^; 3.97 x 10^-8^; 9.92 x 10^-8^; 1.98 x 10^-7^; 9.92 x 10^-7^; 1.98 x 10^-6^; 9.9 x 10^-6^; 1.98 x 10^-5^; 3.97 x 10^-5^; 6.35 x 10^-5^ and 7.94 x 10^-5^ M. Curves 1’ – 17’ correspond to the constant SDS concentration equal to 1 x 10^-8^; 1 x 10^-7^;
1 x 10^-6^; 4 x 10^-6^; 8 x 10^-6^; 1 x 10^-5^; 2 x 10^-5^; 6 x 10^-5^; 1 x 10^-4^; 2 x 10^-4^; 6 x 10^-4^;
8 x 10^-4^; 1 x 10^-3^; 4 x 10^-3^; 6 x 10^-3^, 8 x 10^-3^ and 1 x 10^-2^ M, respectively.


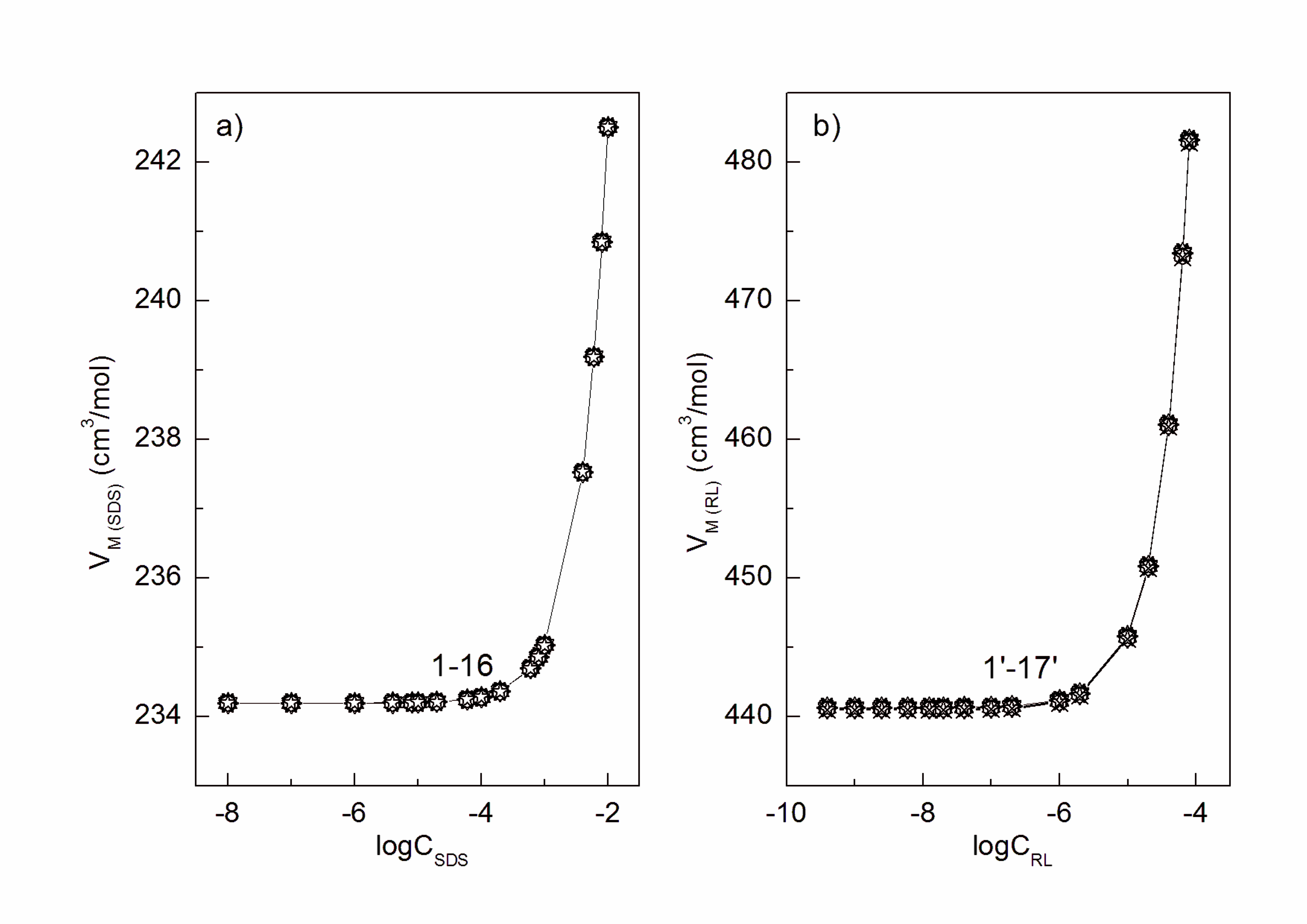


Fig. S11. A plot of the partial molar volume ($\overline{V}_{M}$) of SDS (a) and RL (b) calculated from Eq. S23 vs. the logarithm of surfactant concentration. Curves 1 – 16 correspond to the constant RL concentration ($C_{RL}$) equal to 3.97 x 10^-10^; 9.92 x 10^-10^; 2.48 x 10^-9^;
5.95 x 10^-9^; 1.24 x 10^-8^; 1.98 x 10^-8^; 3.97 x 10^-8^; 9.92 x 10^-8^; 1.98 x 10^-7^; 9.92 x 10^-7^; 1.98 x 10^-6^; 9.9 x 10^-6^; 1.98 x 10^-5^; 3.97 x 10^-5^; 6.35 x 10^-5^ and 7.94 x 10^-5^ M. Curves 1’ – 17’ correspond to the constant SDS concentration equal to 1 x 10^-8^; 1 x 10^-7^;
1 x 10^-6^; 4 x 10^-6^; 8 x 10^-6^; 1 x 10^-5^; 2 x 10^-5^; 6 x 10^-5^; 1 x 10^-4^; 2 x 10^-4^; 6 x 10^-4^;
8 x 10^-4^; 1 x 10^-3^; 4 x 10^-3^; 6 x 10^-3^, 8 x 10^-3^ and 1 x 10^-2^ M, respectively.
